# Neuregulin1 Nuclear Signaling Influences Adult Neurogenesis and Regulates a Schizophrenia Susceptibility Gene Network within the Mouse Dentate Gyrus

**DOI:** 10.1523/JNEUROSCI.0063-24.2024

**Published:** 2024-08-30

**Authors:** Prithviraj Rajebhosale, Alice Jone, Kory R. Johnson, Rohan Hofland, Camille Palarpalar, Samara Khan, Lorna W. Role, David A. Talmage

**Affiliations:** ^1^Genetics of Neuronal Signaling Section, National Institute of Neurological Disorders and Stroke, National Institutes of Health, Bethesda, Maryland 20892; ^2^Graduate Program in Neuroscience, State University of New York at Stony Brook, Stony Brook, New York 11794; ^3^Bioinformatics Core, National Institute of Neurological Disorders and Stroke, National Institutes of Health, Bethesda, Maryland 21042; ^4^Undergraduate Biology, Stony Brook University, Stony Brook, New York 11794; ^5^Circuits, Synapses, & Molecular Signaling Section, National Institute of Neurological Disorders and Stroke, National Institutes of Health, Bethesda, Maryland 20892

**Keywords:** dentate gyrus, ErbB4, gamma secretase, neuregulin1, neurogenesis, schizophrenia

## Abstract

Neuregulin1 (Nrg1) signaling is critical for neuronal development and function from fate specification to synaptic plasticity. Type III Nrg1 is a synaptic protein which engages in bidirectional signaling with its receptor ErbB4. Forward signaling engages ErbB4 phosphorylation, whereas back signaling engages two known mechanisms: (1) local axonal PI3K-AKT signaling and (2) cleavage by γ-secretase resulting in cytosolic release of the intracellular domain (ICD), which can traffic to the nucleus (Bao et al., 2003; Hancock et al., 2008). To dissect the contribution of these alternate signaling strategies to neuronal development, we generated a transgenic mouse with a missense mutation (V_321_L) in the Nrg1 transmembrane domain that disrupts nuclear back signaling with minimal effects on forward signaling or local back signaling and was previously found to be associated with psychosis (Walss-Bass et al., 2006). We combined RNA sequencing, retroviral fate mapping of neural stem cells, behavioral analyses, and various network analyses of transcriptomic data to investigate the effect of disrupting Nrg1 nuclear back signaling in the dentate gyrus (DG) of male and female mice. The V_321_L mutation impairs nuclear translocation of the Nrg1 ICD and alters gene expression in the DG. V_321_L mice show reduced stem cell proliferation, altered cell cycle dynamics, fate specification defects, and dendritic dysmorphogenesis. Orthologs of known schizophrenia (SCZ)-susceptibility genes were dysregulated in the V_321_L DG. These genes coordinated a larger network with other dysregulated genes. Weighted gene correlation network analysis and protein interaction network analyses revealed striking similarity between DG transcriptomes of V_321_L mouse and humans with SCZ.

## Significance Statement

Synaptic contact is predicted to be a regulator of the generation of nuclear signaling by Nrg1. Here we show that a schizophrenia (SCZ)-associated mutation in Nrg1 disrupts its ability to communicate extracellular signals to the neuronal genome that results in altered expression of a gene network enriched for orthologs of SCZ-susceptibility genes. The striking overlap in functional and molecular alterations between a single rare homozygous missense mutation (V_321_L) and SCZ patient data (complex polygenic and environmental burden) underscores potential convergence of rare and common variants on the same cellular and molecular phenotypes. Furthermore, our data indicate that the evolutionarily conserved gene networks that form the basis for this risk are necessary for coordinating neurodevelopmental events in the dentate gyrus.

## Introduction

The Neuregulin 1 (Nrg1)-ErbB4 signaling axis is an important regulator of synapse formation and function throughout the nervous system (Harrison and Weinberger, 2005; Walsh et al., 2008; Agarwal et al., 2014). Type III Nrg1, located presynaptically, participates in bidirectional signaling, i.e., acting as both a ligand and a receptor for ErbB4 (Bao et al., 2003; Talmage, 2008). Upon binding ErbB4, the Type III Nrg1 C-terminal intracellular domain (ICD) can be liberated into the cytosol from the membrane by an intramembrane proteolytic event mediated by the γ-secretase enzyme complex. Following this cleavage, the ICD translocates to the nucleus where it can function as a transcriptional regulator (TR; nuclear back signaling; Bao et al., 2003). Additionally, binding of ErbB4 can also induce local activation of PI3K-AKT signaling (Hancock et al., 2008). Thus, activation of Type III Nrg1 by ErbB4 can induce two distinct modes of signaling. Cortical neurons cultured from Type III Nrg1 KO mice show deficits in axonal and dendritic outgrowth and branching, which were rescued by reexpression of full-length (FL) Type III Nrg1 protein (Chen et al., 2010). However, nuclear back signaling-defective forms of Type III Nrg1, lacking the nuclear localization sequence (NLS) or mutations in the γ-secretase cleavage site, were only able to rescue axonal growth and not dendritic growth (Chen et al., 2010). In line with this, Type III Nrg1 hypomorphic mice showed reduced hippocampal dendritic spine density. Overexpression of the nuclear Nrg1 ICD resulted in increased dendritic spine density in cultured hippocampal neurons, while overexpression of Nrg1 ICD lacking the NLS did not (Chen et al., 2010; Fazzari et al., 2014). Type III Nrg1 heterozygous mice also show other endophenotypes of schizophrenia (SCZ) such as prepulse inhibition (PPI) deficits, increased ventricle volume, and altered hippocampal activity and connectivity (Wolpowitz et al., 2000; Chen et al., 2010; Nason et al., 2011) indicating a possible connection between developmental Nrg1 back signaling and cellular and behavioral endophenotypes of severe neurodevelopmental disorders such as SCZ.

A missense mutation in *Nrg1* resulting in a single amino acid substitution in a transmembrane valine (to leucine; rs74942016—results in Val→Leu at Position 321 in Type III Nrg1 β1a) was found to be associated with SCZ (and more strongly with psychosis) in a Costa Rican population (Walss-Bass et al., 2006). Intriguingly, γ-secretase cleaves between cysteine 320 and valine 321, and leucine substitution for V_321_ results in impaired cleavage and reduced nuclear translocation of the ICD (Chen et al., 2010; Fleck et al., 2016). This presents the intriguing possibility that loss of a specific signaling modality within this multifunctional protein might contribute to some features of neurodevelopmental disorders such as SCZ.

To isolate the effects of nuclear back signaling by the Nrg1 ICD on neuronal development and transcriptomic regulation, we generated transgenic mice harboring the V_321_L mutation in Nrg1. The dentate gyrus (DG) serves as the gate for incoming information into the hippocampus. Neuronal development is an ongoing process in the DG, owing to the presence of a neurogenic niche in the postnatal brain, thereby allowing us to broadly sample effects on various stages of neuronal development from neuronal fate commitment to maturation. Thus, we chose the DG as a candidate region to begin investigating the effects of impaired γ-secretase processing of Nrg1.

## Methods and Materials

### Experimental model and subject details

Male and female mice ages Embryonic Day (E)18.5–6 months were used. Animals were housed in a 12 h light/dark cycle (lights on from 7 A.M. to 7 P.M.) environment that was both temperature and humidity controlled. Animals had *ad libitum* access to food and water. All animal care and experimental procedures were approved by the Institutional Animal Care and Use Committee of the State University of New York Research Foundation at Stony Brook University (Protocol Number 1618) and NINDS, National Institutes of Health (Protocol Number 1490).

For studies profiling proliferating cells in the DG, mice around the age of Postnatal Day (P)21 were used to avoid a “floor effect” due to decline in proliferating cells with age (Ben Abdallah et al., 2010). Mice aged ∼2 months were used for counting Dcx+ cells as Dcx immunoreactivity is still fairly abundant in the DG at this age. For studies of cell cycle dynamics, we used mice around the age of P90 as death of proliferating cells is high in younger mice and stabilizes around this age thereby allowing us to detect the proliferative state of the same cell using EdU and Ki67 (Ansorg et al., 2012). For the morphological studies, we intended to profile enduring changes in mature GCs. The bulk of granule cells (GCs) undergo a relatively “sudden” maturation (profiled by gene expression changes) around the third postnatal week (Hochgerner et al., 2018). Furthermore, complete morphological maturation of a GC takes ∼1 month (Sun et al., 2013). Thus, to escape the dynamic period of early maturation and presence of large numbers of immature neurons in the GC layer (GCL), we assessed morphology of GCs after 2 months of age.

### Genotyping

Genotypes were determined by PCR using the following primers:

NDEL1: Forward primer 5′- GGTGATCCCATACCCAAGACTCAG -3′
NDEL2: Reverse primer 5′- CTGCACATTTATAGAGCATTTATTTTG -3′

### PPI

Male and female mice between the ages of 1 and 3 months were used. Testing was performed in dark sound-attenuated boxes, between the hours of 12 P.M. and 4 P.M.; male and female mice were tested separately, and all equipment was thoroughly cleaned between mice using Clorox Fuzion cleaner allowing ∼2 min contact time with the equipment. All testing was performed using the SR-LAB Startle Response System (San Diego Instruments). Mice were transported to the behavior testing facility and held in a holding room. Mice were acclimated to the holding room for at least 5 min and then to the startle chambers by placing them into the startle chamber and exposing them to constant background white noise set at 65 dB for 5 min for 3 d. On the third day, mice also underwent an input–output curve calibration where they were exposed to startle stimuli increasing in 5 dB increments from 70 to 120 dB; an input–output curve was plotted for each mouse to identify the saturation of the startle response. All mice tested showed maximal startle responses at 115 dB which was used as the startle stimulus in the habituation and PPI trials. On the fourth day, mice were once again exposed to 65 dB background white noise for 5 min after which they entered “Block 1” consisting of sixty 20 ms 115 dB startle stimuli with an intertrial interval of 20 s. Block 1 continued into “Block 2” consisting of 10 trials each of the following combinations: 4 ms 68/70/75/85 dB prepulses followed by a 115 dB startle stimulus separated by an interval of either 30 or 100 ms. Twenty 20 ms startle stimuli (115 dB) were interspersed between these trials. Intertrial intervals (ITIs) were randomized using the ITI function within the SR-LAB software, and the trial order was randomized using an atmospheric noise randomizer (www.random.org/lists). PPI is known to improve upon repeat testing, stabilizing thereafter (Valsamis and Schmid, 2011). Thus, on the fifth day, mice were once again exposed to Blocks 1 and 2. Responses from Day 5 were used as the outcome measures. Additionally, fecal pellets were counted after every session and recorded for each mouse.

#### PPI calculation

Startle responses were identified as the maximal voltage deflection recorded from the startle box during the 20 ms startle pulse delivery. An average of the responses to the 20 startle stimuli alone delivered in Block 2 were used as the baseline startle measurement to calculate %PPI. %PPI was calculated as [(average startle response during PPI trial / baseline startle response) * 100]. All %PPI values were capped at a lower limit of 0% (thus, any negative PPI was set to and reported as 0% PPI).

### Antibody generation

Antibodies recognizing the Nrg1 ICD were raised in rabbits using the immunizing peptide: DEE(pY)ETTQEYEPAQEP by Biomatik. Antibodies recognizing the phosphorylated peptide DEE(pY)ETTQEYEPAQEP versus the unphosphorylated peptide DEEYETTQEYEPAQEP were purified by affinity chromatography. A cocktail of antibodies referred to as anti-Nrg1 ICD_DAT_ was used for staining cells comprised of antibodies against the phosphorylated and nonphosphorylated peptides. The phospho-specific antibodies were developed for a different study. Antibodies were validated for immunofluorescence and immunoblotting by comparing samples from N2A cells and N2A cells transfected with a Type III Nrg1-YFP construct. Detectable staining of N2A cells in ICC experiments and unique bands corresponding to the appropriate molecular weight was only observed in transfected samples.

### Subcellular fractionation

For each experiment, the cortex and hippocampus were pooled by genotype for nuclear fractionation. P86–108 animals were deeply anesthetized with isofluorane and decapitated, and the cortex and hippocampus were isolated on ice and homogenized with Dounce homogenizer on ice in 5 ml nuclear fractionation buffer (60 mM KCl, 15 mM NaCl, 15 mM Tris–HCl, 1 mM EDTA, 0.2 mM EGTA, 15 mM 2-mercaptoethanol, 0.5 mM spermidine, 0.15 spermine, 0.32 M sucrose, cOmplete ULTRA tablets protease inhibitor cocktail), pH7.6. Samples were first homogenized with the loose pestle checking for single, dispersed cells after every 10 strokes using a hemocytometer. Samples were then homogenized with the tight pestle checking for dissociated nuclei after every 10 strokes. Samples were centrifuged at 1,000 × *g* for 10 min at 4°C. The supernatant (S1) was preserved for further processing. The pellets were resuspended in nuclear fractionation buffer with 0.1% Triton X-100 and incubated on ice for 10 min and then pelleted at 1,000 × *g* for 10 min at 4°C. Pellets were resuspended in 500 µl nuclear fractionation buffer, layered over 500 µl of a 1 M sucrose (in nuclear fractionation buffer) cushion, and centrifuged at 1,000 × *g* for 10 min at 4°C. Pellets were resuspended in 1 ml nuclear fractionation buffer.

To isolate the membrane fractions, we centrifuged the fraction S1 at 10,000 × *g* for 10 min at 4°C to remove mitochondria, lysosomes, and peroxisomes. The supernatant (S2) was collected and centrifuged at 100,000 × *g* for 1 h at 4°C. The pellet was resuspended in 1 ml subcellular fractionation buffer [SFB; 250 mM sucrose, 20 mM HEPES, 10 mM KCl, 1.5 mM MgCl_2_, 1 mM EDTA, 1 mM DTT (add fresh before use), cOmplete ULTRA tablets protease inhibitor cocktail] and passed through a 25 gauge needle 10 times, and the sample was centrifuged at 100,000 × *g* for 45 min at 4°C. The supernatant was discarded, and the pellet was resuspended in 200 µl SFB.

Laemmli buffer was added to each sample and boiled at 95°C for 5 min. Denatured samples were stored overnight at −20°C. Protein analysis was then performed by immunoblot.

### Immunoblotting

Samples were electrophoresed on 8% SDS-PAGE gel at 90 V for 1.5 h at room temperature (RT). Proteins were transferred onto nitrocellulose membranes via wet transfer performed at 4°C at 100 V for 1 h. Membranes were blocked in Odyssey Blocking Buffer (LI-COR Biosciences 927-40000) for 1 h at RT with gentle agitation. Membranes were then incubated overnight on a rocker at 4°C with primary antibodies diluted in 50% blocking buffer. The next day, membranes were washed three times with PBS-T (phosphate-buffered saline + 0.1% Tween 20) and incubated with secondary antibodies (diluted in 50% blocking buffer) for 1 h at RT with gentle agitation. After three washes in PBS-T, membranes were imaged using the LI-COR Odyssey Infrared Imaging System.

#### Cell culture

*Primary neuronal culture*: P4–5 wild-type (WT) and V_321_L mice were rapidly decapitated, and their brains were transferred to ice-cold Hibernate-A. Hippocampi were dissected out and transferred to Hibernate-A (Thermo Fisher Scientific) on ice. Hibernate solution was removed and replaced with activated and filtered Papain (Sigma-Aldrich Lot Number SLBZ8588) dissolved in Hibernate-A. Hippocampi were digested for 15 min in a 37°C water bath gently inverting the tube every 5 min. Papain solution was removed, and the digested tissue was washed thoroughly (four times) with wash solution [Hibernate-A + 10%FBS + 100U DNase I (STEMCELL Technologies)] by resuspending the tissue in wash solution and allowing it to settle before replacing wash solution. Forty micrometer cell strainers were placed over 50 ml conical tubes and wet with plating medium (Neurobasal + B27 + Glutamax + Pen/Strep + HEPES), and flow through was discarded. Wide and narrow bore fire-polished Pasteur pipettes were prewet with FBS and were used to triturate the tissue into a single-cell suspension. The cell suspension was applied to the prewet cell strainer and flow through was collected and analyzed for live/dead cells using Trypan blue on the Countess II automated cell counter. Approximately 100 kcells/well were plated into 24-well plates containing 12 mm poly-D-lysine + laminin-coated coverslips in a total volume of 250 µl/well. Neurons were left overnight to adhere, and 250 µl fresh plating medium was added the following day to each well. Fifty percent of the total media was replaced every other day until experimental time points.

For cortical cultures, cortices were isolated from E18.5 embryos.

*Cell line culture and transfection*: Neuro2A cells (ATCC) were maintained in DMEM + 10%FBS + 1:1,000 gentamycin. Cells were passaged on a Mon-Wed-Fri scheduled and were not used beyond 10 passages. Cells were transfected with plasmid DNA using Lipofectamine 2000 following manufacturer guidelines. Plasmid details can be found in Table 1.

**Table 1.** Key resources table

Reagent or resource	Source	Identifier
Antibodies		
Rabbit anti-Neuregulin1 ß1a (1:500)	Santa Cruz Biotechnology	Catalog #sc-348
Rabbit anti-Neuregulin1 ß1a (ICC-1:500, WB-1:1,000)	This paper	N/A
Mouse anti-TuJ (1:1,000)	BioLegend/Covance	Catalog #MMS-435P
Rabbit anti-Ki67 (1:500)	Abcam	Catalog #ab15580
Goat anti-Dcx (1:500)	Santa Cruz Biotechnology	Catalog #sc-8066
Rabbit anti-GFP (1:1,000)	LifeTech	Catalog #A11122
IRDye800-conjugated affinity purified goat anti-rabbit IgG (H&L) (1:20,000)	Rockland Immunochemicals	Catalog #611-132-002
Goat anti-rabbit Alexa Fluor 680 conjugate (1:30,000)	LifeTech	Catalog #A21109
Donkey anti-rabbit Alexa Fluor 488 conjugate (1:1,000)	LifeTech	Catalog #A21206
Donkey anti-rabbit Alexa Fluor 594 conjugate (1:1,000)	LifeTech	Catalog #A21207
Donkey anti-goat Alexa Fluor 488 conjugate (1:1,000)	LifeTech	Catalog #A11055
Donkey anti-mouse Alexa Fluor 488 (1:1,000)	LifeTech	Catalog #A21202
Bacterial and virus strains		
MMLV (VSV-G)-GFP	Dr. Shaoyu Ge	https://www.nature.com/articles/nature04404
Chemicals, peptides, and recombinant proteins		
BenchStable DMEM	Invitrogen	Catalog #A4192101
Glutamax	Invitrogen	Catalog #35050061
Recombinant mouse ErbB4/Her4 Fc chimera protein, CF	R&D Systems	Catalog #4387-ER-050
Papain from papaya latex	Sigma-Aldrich	Catalog #P4762 Lot Number SLBZ8588
BDNF	Invitrogen	Catalog #10908-010
B-27 supplement (50×), serum free	Invitrogen	Catalog #17504044
PFA, 32% *w*/*v* aq. soln., methanol free	Thermo Fisher Scientific	Catalog #473779M
DAPI Fluoromount-G	SouthernBiotech	Catalog #101442-494
Spectral DAPI	Akoya Biosciences	Catalog #FP1490
ProLong Gold Antifade	Thermo Fisher Scientific	Catalog #P36934
cOmplete Protease Inhibitor Cocktail	Sigma-Aldrich	Catalog #11836145001
PhosSTOP	Sigma-Aldrich	Catalog #4906837001
DAPT	CST	Catalog #15020S
RNA*later* Stabilization Solution	Invitrogen	Catalog #AM7020
Hibernate-A medium	Invitrogen	Catalog #A1247501
Opal 570 Reagent Pack	Akoya Biosciences	Catalog #FP1488001KT
Opal 620 Reagent Pack	Akoya Biosciences	Catalog #FP1495001KT
Opal 690 Reagent Pack	Akoya Biosciences	Catalog #FP1497001KT
Deposited data		
RNA-Seq data	https://www.ncbi.nlm.nih.gov/geo/query/acc.cgi?acc=GSE192869 https://www.ncbi.nlm.nih.gov/geo/query/acc.cgi?acc=GSE268856	GEO accession codes: GSE192869 GSE268856
Raw sequencing reads	This paper	(Available on SRA) BioProject ID: PRJNA793574 BioProject ID: PRJNA1119194
Experimental models: cell lines		
Neuro2a (ATCC CCL-131)	ATCC	
HEK293-T	ATCC	
Experimental models: organisms/strains		
Mouse: V321L: Nrg1tm1Dat V/L	This paper	
Oligonucleotides		
Genotyping Fwd Primer: 5′-GGTGATCCCATACCCAAGACTCAG-3′	IDT	
Genotyping Rev Primer: 5′- CTGCACATTTATAGAGCATTTATTTTG -3′	IDT	
RNAscope Probe—Mm-Calb1—*Mus musculus* calbindin 1 (Calb1) mRNA	ACD/Bio-Techne	428,431
RNAscope Probe—Mm-Prox1—*Mus musculus* prospero homeobox 1 (Prox1) mRNA	ACD/Bio-Techne	488,591
RNAscope Probe—Mm-Synpr-C2—*Mus musculus* synaptoporin (Synpr) transcript variant 1 mRNA	ACD/Bio-Techne	500,961
RNAscope Probe—Mm-Ezh2-C3—*Mus musculus* enhancer of zeste homolog 2 *(Drosophila*; Ezh2) transcript variant 1 mRNA	ACD/Bio-Techne	446,611
RNAscope Probe—Mm-Chrm5-C3—*Mus musculus* cholinergic receptor muscarinic 5 (Chrm5) mRNA	ACD/Bio-Techne	495,301
RNAscope Probe—Mm-Cit-C2—*Mus musculus* citron (Cit) mRNA	ACD/Bio-Techne	567,401
Recombinant DNA		
pUX-GFP	Dr. Shaoyu Ge	
pCMV-VSV-G	Dr. Shaoyu Ge	Addgene Plasmid 8454
GP	Dr. Shaoyu Ge	
Type III Nrg1-YFP	Dr. Kevin Czaplinski	
Software and algorithms		
Code for RNA-Seq analysis	https://github.com/RajNINDS/V321L_DG	

#### Stimulation and drug treatments

Recombinant Mouse ErbB4/HER4 Fc Chimera Protein was reconstituted at a final concentration of 0.5 µM and stored at −80°C. Working stocks of 0.1 µM were prepared and stored briefly at −20°C. For stimulation experiments, 20 nM ErbB4 solution was prepared by diluting 0.1 µM stock solution in 125 µl Neurobasal plating medium/well.

Neurons were washed by gently swirling the plate and aspirating out all the media and near-simultaneously replacing with 125 µl of fresh prewarmed plating media and equal volume of drug concentrations; (pre)treatment periods are listed in Table 2.

**Table 2.** Pharmacological treatment parameters

Drug name	Manufacturer	Concentration	Treatment time
DAPT	CST	20 µM	24 h, 45 min
sErbb4	R&D Systems	20 nM	15–20 min
WM	Sigma-Aldrich	500 nM	45 min
L685458	Sigma-Aldrich	5 mM	45 min

Cell cultures were treated with different pharmacological agents: N-[N-(3,5-Difluorophenacetyl)-Lalanyl]-S-phenylglycine t-butyl ester (DAPT- gamma secretase inhibitor), recombinant soluble ectodomain of human ErbB4 (sErbB4), Wortmannin (WM- PI3K inhibitor), L685458 (gamma secretase inhibitor). The treatment time for DAPT indicates a 24 h pre-treatment, followed by an acute 45 min treatment.

#### Immunocytochemistry

Neurons were fixed for 5 min at RT in 4% paraformaldehyde (PFA) by adding equal volume of 8% PFA to neurobasal in the wells. The entire solution was replaced with fresh 4% PFA, and neurons were fixed further for 10 min at RT. Coverslips were washed three times for 5 min each in 1× PBS-G (PBS + 0.1 M glycine), incubating in the final wash for 10 min. Neurons were then permeabilized in 0.1% Triton X-100 in 1× PBS for 15 min at RT. Coverslips were washed three times for 5 min each with 1× PBS-G and incubated in blocking solution (10% normal donkey serum-NDS) for 30 min. After blocking, coverslips were incubated with primary antibodies prepared in 1% NDS overnight at 4°C. Coverslips were washed thoroughly with 1× PBS at RT three times for 5 min each and were then incubated with secondary antibodies prepared in 1%NDS + 0.1%Triton X-100. Coverslips were washed thoroughly with 1× PBS at RT three times for 5 min each and mounted in mounting medium containing DAPI (DAPI Fluoromount-G, SouthernBiotech). Antibodies used and concentrations can be found in Table 1.

#### Tissue processing and immunohistochemistry

Animals were deeply anesthetized with isofluorane and transcardially perfused with 4% PFA in PBS. Brains were harvested and postfixed overnight at 4°C in 4% PFA in PBS. Brains were transferred to 30% sucrose in PBS and incubated at 4°C with agitation until they sank. Brains were embedded in optimal cutting temperature compound, flash frozen, and stored at −80°C. The 20–50 µm sections were obtained serially over the entire dorsoventral extent of the hippocampus, and each slide collected one of every eight (20 µm) or one of every four sections (50 µm). Sections were dried at RT overnight prior to immunohistochemistry.

Antigen retrieval was performed for Ki67 staining by immersing sections in antigen retrieval buffer (10 mM sodium citrate, 0.05% Tween 20), pH 6.0, at 100°C for 15 min. Sections were then allowed to cool to RT and neutralized in 0.1 M borate buffer, pH8.5, for 10 min at RT. After three 5 min washes in PBS, sections were incubated in blocking buffer (5% normal donkey serum, 0.1% Triton X-100 in PBS) for 1 h at RT. Sections were then incubated with primary antibodies diluted in blocking buffer for 48 h at 4°C in a humidity chamber. After three 5 min washes in PBS, sections were then incubated with secondary antibodies diluted in blocking buffer for 1 h at RT. Sections were dehydrated in 70% ethanol for 5 min and treated with autofluorescence eliminator reagent (Millipore Sigma 2160). Number 1.5 glass coverslips were then mounted on the slides with DAPI Fluoromount-G and allowed to dry overnight at RT.

Antibodies used and concentrations can be found in Table 1.

#### Fluorescent in situ hybridization (FISH)

Brains were fixed and cryosectioned as described earlier. Fifteen micrometer sections were prepared with two coronal sections on a slide. FISH was performed per manufacturer’s instructions using the RNAscope v2 fluorescent multiplex assay [Advanced Cell Diagnostics (ACD)/Bio-Techne]. Details on the probes used can be found in Table 1.

#### Imaging and image analysis

To image Nrg1 ICD puncta in the nucleus, imaging was done on the Zeiss LSM800 confocal microscope using the 63× oil immersion objective. All samples were imaged using identical parameters. Neurons to analyze were identified by expression of Type III Nrg1 (CRD+), and their nuclei were imaged at 2.5× digital zoom with 1 µm *z*-steps.

Image processing and analysis were performed using ImageJ (Fiji). Briefly, the center of the nucleus was identified, and one *z*-section above and below it was flattened via maximum intensity projection. DAPI-stained nuclei were outlined, and the enclosed area was measured and noted. ICD clusters were manually counted using the Cell Counter plugin. Density of clusters was calculated by dividing number of clusters by the nuclear area.

Images examining Type III Nrg1 expression profile in the DG and images used for Ki67+ total cell counts in P21 animals were done using the Optical Fractionator probe on the Stereo Investigator software (MBF Bioscience) coupled to live imaging on an epifluorescent microscope. Section sampling fraction was one every eighth section, the counting frame size used was 100 µm × 100 µm, and guard zone height used was 10 µm. All sample counts achieved a Gundersen coefficient of error of <0.1.

Dcx+ cell density counts were acquired on Olympus FLV1000 confocal microscope at 20× magnification with 3 µm *z*-steps. Images were analyzed in ImageJ using the Cell Counter plugin. Only cells located in the subgranular zone of the DG were counted. Total cell counts for each animal were divided by the total DG volume counted to obtain cell density. For each animal, 12–14 coronal sections were sampled along the entire dorsoventral extent of the DG.

EdU labeling counts were done on images acquired using Olympus VS-120 microscope at 20× magnification. Images were acquired at 1 µm *z*-steps of the entire DG hemisection. Images were analyzed in ImageJ using the Cell Counter plugin. Total cell counts for each animal were divided by the total DG volume counted to obtain cell density. For each animal, 12–14 coronal sections were sampled along the entire dorsoventral extent of the DG.

RNAscope FISH sections were imaged on a Nikon Ti2 spinning disk confocal microscope using a 40× silicone immersion objective. Images were acquired at 1 µm *z*-steps of the DG. The images were *z*-projected, and an area in the molecular layer was identified to assess background staining. The fluorescence intensity of the background was measured and then subtracted using the Math > Subtract function on Fiji (ImageJ). Background subtracted images were then used to delineate an ROI consisting of the GCL using DAPI staining as a guide. The “IntDen” measurement within the GCL was recorded. Two hippocampal sections (i.e., four hippocampi) at different anterior–posterior locations (consistent between mice) were quantified per mouse and averaged.

### Golgi impregnation, sampling criteria, imaging, and analysis

P60 animals were deeply anesthetized with isofluorane, and brain samples were harvested quickly on ice and rinsed with ice-cold PBS. The brain samples were then treated with the FD Rapid GolgiStainTM Kit (FD NeuroTechnologies, catalog #PK401) per manufacturer’s guidelines. One hundred micrometer brain sections were collected using a vibratome and dried overnight in the dark at RT. Sections were then dehydrated in progressively increasing concentrations of ethanol solution (50% → 75% → 95%), then xylene, and coverslipped with Permount solution.

Imaging of Golgi-impregnated neurons in the DG was done on the Olympus VS-120 microscope at 60× magnification. Individual neurons were selected for imaging based on the following **criteria**: (1) the neuron’s cell body resided in the GCL in the intermediate section of the superior blade, (2) the soma morphology assumed the typical GC “tear drop” morphology, (3) the dendritic arbor originated from a single primary dendrite and assumed a cone-shaped morphology, and (4) the dendritic arbor was not obstructed by other cell bodies or dendrites. Eleven neurons from five WT animals and 13 neurons from five homozygous mutants were ultimately selected for analysis. Dendritic arbor tracings, sholl analysis, and branching analysis were done on the NeuronStudio software.

### GFP-expressing retrovirus production, injection, imaging, and analysis

GFP retrovirus was prepared by cotransfection of pUX-GFP, VSV-G, and GP constructs (kindly provided by the laboratory of Shaoyu Ge; previously described in Ge et al., 2006) into HEK293 cells. Viral supernatant was collected, the retrovirus was concentrated by ultracentrifugation, and the viral pellet was resuspended in sterile PBS.

Following anesthetization, ∼P90 animals were stereotaxically injected bilaterally in dorsal DG (−2.0 mm A/P, +1.6 mm M/L, −2.5 mm D/V) and ventral DG (−3.0 mm A/P, +2.6 mm M/L, −3.2 mm D/V). The injection volume was 0.25 µl/site. Following surgery, animals were administered 0.03 ml ketorolac (3 mg/ml) for every 10 g body weight.

Fourteen days following stereotaxic viral injections, animals were killed, and brain samples were harvested as described previously for tissue preparation. Fifty micrometer floating coronal sections were obtained, and each set of sections contained one of every sixth section of the hippocampus along its dorsoventral extent.

Analysis was done in real time while imaging on the Zeiss Axio Imager A1 fluorescent microscope using a 40× oil immersion objective lens. The identity of GFP+ cells were determined by a combination of morphology, Dcx immunoreactivity, and Nissl staining. Neural progenitors (NPs) were identified by short, tangential processes and the lack of radially oriented processes, with or without Dcx immunoreactivity. Immature GCs (iGCs) were identified by their radially oriented neurites, Dcx+ and Nissl+ staining. Astrocytes were identified by their star-like morphology, extensive branch ramifications, and lack of Dcx immunoreactivity and Nissl staining.

### EdU labeling and tissue processing (cell cycle exit, length, and survival)

To measure cell cycle reentry, we injected EdU (50 µg/g body weight) intraperitoneally in animals at ∼P90. Twenty-four hours after EdU injection, animals were killed, and brain samples were harvested and prepared as described in the previous section. To visualize EdU incorporation, we used the Click-iTTM EdU Cell Proliferation Kit (Thermo Fisher Scientific, catalog #C10338) according to manufacturer’s guidelines, followed by immunohistochemistry as described in the previous section.

To measure cell cycle length, we injected EdU (50 µg/g body weight) intraperitoneally in animals at P21–30. Thirty minutes after EdU injection, animals were killed, and brain samples were harvested and prepared as described in the cell cycle reentry experiment. To measure newborn cell survival, we injected three pulses of EdU (50 µg/g body weight) spaced 24 h apart intraperitoneally in animals at ∼P90. Animals were killed 30 d later, and brain samples were harvested.

### RNA sample prep and sequencing

All tools, glassware, gloves, and surfaces were cleaned with ethanol and RNaseZAP (Invitrogen) according to manufacturer guidelines.

Mice were anesthetized using isoflurane. Mice were then placed in a shallow ice trough and rapidly perfused with 10 ml ice-cold 1× PBS (total time required from induction of anesthesia to end of perfusion was ∼5 min). The brain was immediately removed and transferred to a prechilled stainless-steel adult mouse brain matrix placed in ice to make 0.3 mm coronal sections (∼2 min) using prechilled blades. The sections were immediately transferred to ice-cold Hibernate-A in a prechilled glass Petri dish on ice. DG was visually identified and dissected out from hippocampal sections to isolate the upper and lower blades along with adjoining regions of the hilus and part of the molecular layer (Fig. 5*A*). The microdissections took no >3 min per brain. Dissected DG bits were transferred to 1 ml RNAlater in a microcentrifuge tube placed in ice and were left rotating at 4°C overnight. The following day, the tissue was extracted from RNAlater solution, flash frozen using dry ice and 100% ethanol, and stored at −80°C until RNA extraction.

RNA was extracted from flash frozen DG bits and cultured neurons using the RNeasy Micro Kit (Qiagen) with on-column DNase treatment following manufacturer guidelines. RNA was poly-A selected, and RNA-Seq libraries were prepared using NEBNext Library Prep Kit for Illumina (New England Biolabs E7760). A MiSeq shallow sequencing run was performed after library preparation to balance the sample loading process on the deep sequencer. Sequencing was performed using NSQ 500/550 Hi Output KT v2.5 (150 CYS; Illumina) to obtain 75 bp paired end reads. The estimated read depth was ∼33.33 million reads/DG sample.

### Differential gene expression (DEG) analysis

QC inspection was performed on sequencing data using a combination of FastQC, MultiQC, and Fastx_Screen. Next, adaptor sequences were clipped and low-quality and/or nucleotide-biased positions in sequences were trimmed using the Trimmomatic; QC inspection of the post clipped and trimmed sequences was repeated. Clipped and trimmed sequences were aligned to the mouse reference genome (mm10) using the HISAT2 tool, and QC inspection of the alignments was performed using the RSeqC. Transcript assembly was performed using StringTie which included the discovery of any novel genes:isoforms. QC inspection of the assembly was performed using the gffcompare tool. To enumerate both gene-level and transcript-level expression from the assembly output, IsoformSwitchAnalyzeR package supported in R was used. For differential analysis of the enumerated gene-level expression, commands and functions supported in R were used. Briefly, expression per sample was first within sample normalized via cpm (counts per million) then pedestalled by 2 by taking the log2 transformation. To correct for differences in spread and location, required before testing, we performed cross-sample normalization using Cyclic Lowess. Expression after normalization was inspected by cov-based PCA scatterplot to identify any outliers. Using this one sample was identified as an outlier (WT female). This sample was further inspected for coverage using a set of housekeeping genes in mm10 and was found to be 5′ degraded and as such was excluded from further analysis. Other samples were reinspected for coverage and were found to pass QC. Normalization for the gene-level expression was repeated after outlier removal, and sequence batch was globally corrected for via the Limma tool. Using the noise model plot generated from Limma, we identified a mean batch-corrected normalized expression value of 2.25 that was then used to filter genes and transcripts not having at least one sample greater than this value while also flooring values to 2.25 for surviving genes and transcripts if less than this value. These filter-floored values for surviving transcripts were tested for differential expression between WT and V_321_L samples via ANCOVA controlling for sequence batch and biological sex. Dysregulated genes were identified as those having a *p* value of <0.05 or <0.1 (colored differently on volcano plot) corrected for batch and/or sex and a minimum fold change (FC) of 1.25 in either direction. Dysregulated genes were then used to perform enrichment analyses using Ingenuity Pathway Analysis (IPA; Qiagen) and Enrichr (Chen et al., 2013; Kuleshov et al., 2016; Xie et al., 2021). Weighted gene correlation network analysis (WGCNA) was conducted using the R package (detailed code can be found on the Github link provided below).

### Network analyses

SCZ-related enriched terms were combined into a single pathway in IPA yielding 67 genes. These 67 genes were selected as seeds, and a network was grown using the Build > Grow tool in IPA. The following criteria were set to grow the network: with direct interactions only using the DEG list from the V_321_L versus WT dataset, using all data sources within the IPA knowledge base, for miRNAs we selected only those that were experimentally observed. For data from cell lines and tissues, we selected only data from CNS cell lines, neuroblastoma cell lines, and neurons using the stringent filter setting to ensure that two nodes will only be connected if the genes are expressed in neural cells. We also excluded “correlation” and “membership” as relationship criteria for connecting nodes leaving behind direct physical or signaling interactions.

Genes from WGCNA modules were used to generate PINs using STRING (Szklarczyk et al., 2023). We adjusted the settings to only include interactions from experiments, databases, and gene fusions. The minimum required interaction score was set to 0.9 (highest stringency) and disconnected nodes were removed.

Networks were exported to Cytoscape, and hub gene analysis was performed using the CytoHubba plugin.

### Statistical analysis

Statistical analysis was performed using Prism (GraphPad). Normality was assessed using Shapiro–Wilk and Kolmogorov–Smirnov tests. If data failed either test, nonparametric stats were used. *p* values were corrected for multiple comparisons as necessary using Bonferroni’s (parametric) post hoc test.

For sholl analysis, we followed published guidelines to avoid faulty inferences in sholl analyses (Wilson et al., 2017). Sholl analysis data did not pass Shapiro–Wilk normality test; however, given that measurements were made from multiple neurons from the same animal pooled with neurons across animals, we could not use the Wilcoxon rank sum test given the assumption of independence of variables. To address this, we transformed the data by taking the square-root of the measurements and used a repeated-measure (RM) two–way ANOVA with a mixed effect model. To control for false discovery rate (FDR) and Type 1 error inflation, we corrected the *p* values for multiple comparisons using the original Benjamini–Hochberg FDR procedure.

## Results

### Generation of the Nrg1 V_321_L mutant mouse to study γ-secretase-mediated Nrg1 signaling

**Figure 1.**
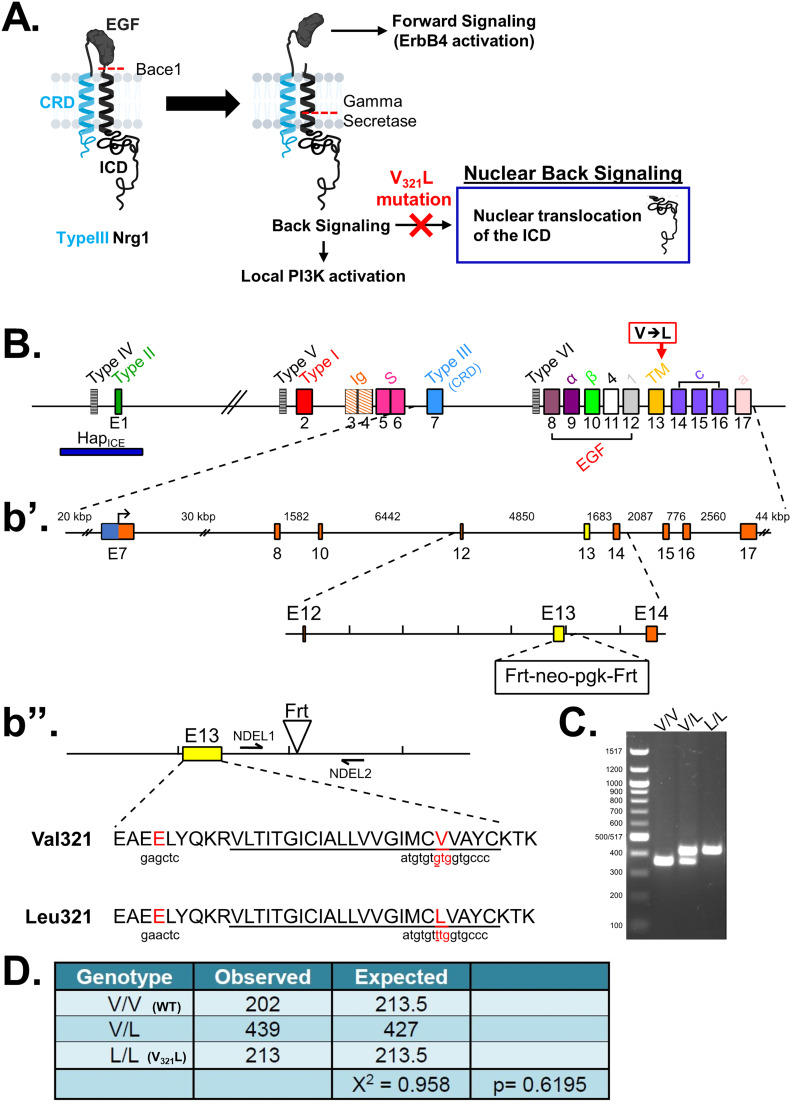
Generation of Nrg1 V_321_L knock-in mice. ***A***, A schematic of the different modes of signaling engaged by Type III Nrg1. Cleavage by Bace1 is an essential step to generate a substrate for γ-secretase. The extracellular EGF-like domain of Nrg1 can interact with ErbB4 on neighboring cells to engage the canonical forward signaling by activating ErbB4. ErbB4 interaction can also result in activation of back signaling by Nrg1. Local back signaling engages PI3K signaling at the membrane, whereas cleavage by γ-secretase results in liberation of the C-terminal ICD, which can translocate to the nucleus–nuclear back signaling. The V_321_L mutation blocks cleavage by γ-secretase and is predicted to disrupt nuclear back signaling. ***B***, A schematic diagram of the *Nrg1* genomic structure. The *Nrg1* gene encodes six families of isoforms as a result of alternative promoter usage. Types 1, 2, 4, and 5 contain Ig-like domains (encoded by Exons 3 and 4). Exon 7 is the unique 5′ coding exon for Type III Nrg1, encoding an N-terminal, cysteine-rich transmembrane domain. Exon 8, in combination with Exon 9 or 10, and various combinations of Exons 11, 12, and 13 encode an EGF-like domain common to all Nrg1 isoforms. Exon 13 encodes a common C-terminal transmembrane domain. The C-terminal ICD is encoded by Exons 14–17. The missense SNP that results in a valine-to-leucine substitution in the C-terminal transmembrane domain is shown above the gene. ***b*′**, Schematic diagram of the BAC clone used for generating the target construct corresponding to a region of the Nrg1 gene that comprises the entire Type III coding region. Below the BAC clone is a diagram of the targeting construct, including a 5.8 kbp left homology arm, a neoR cassette in the antisense orientation flanked by Frt sites, and a 2.5 kbp right homology arm. ***b*″**, Diagram of WT and mutant alleles (the mutant allele following flippase removal of the neo cassette). The C-terminal transmembrane domain sequence is underlined. Black arrows labeled “NDEL1” and “NDEL2” indicate approximate genomic locations of genotyping primers. ***C***, An example genotyping gel of offspring from a het × het cross illustrating a WT, heterozygote and homozygote. ***D***, The mutant mouse line was maintained by breeding heterozygotes. The genotype of pups from >100 litters was analyzed for deviation from the expected 1:2:1 ratio. No deviation was found. Mice with the genotype V/V are referred to as WT in the manuscript and L/L are referred to as V_321_L as denoted in parenthesis.

Nrg1 proteins can back signal through at least two different mechanisms: (1) local activation of PI3K-Akt signaling and (2) nuclear translocation of a free C-terminal fragment generated by γ-secretase cleavage. The V_321_L mutation disrupts the preferred γ-secretase cleavage site (Fig. 1*A*). To examine the effect of the V_321_L substitution on Nrg1 signaling, we generated a knock-in germline mutation in mice. The V_321_L mutation (gtg→ttg) was introduced into C57Bl6 embryonic stem cells using a bacterial artificial chromosome (BAC) construct (Fig. 1*B–D*). Heterozygotes were interbred, and offspring were born at the expected Mendelian ratios (Fig. 1*D*). Homozygous V_321_L mutants were viable and fertile, with no outward morphological or growth abnormalities. Homozygous animals were able to interbreed and yielded normal-sized litters with offspring that appeared healthy. The V_321_L mutation was also viable on a Type III Nrg1 knock-down background, as a *Nrg1*^L/L^ × Type III *Nrg1*^+/−^ cross yielded both *Nrg1*^V/L^ and *Nrg1*^L/−^ offspring.

### Neurons from Nrg1 V_321_L mutant mice show diminished nuclear back signaling and lack of dendritic growth in response to stimulation with soluble ErbB4

**Figure 2.**
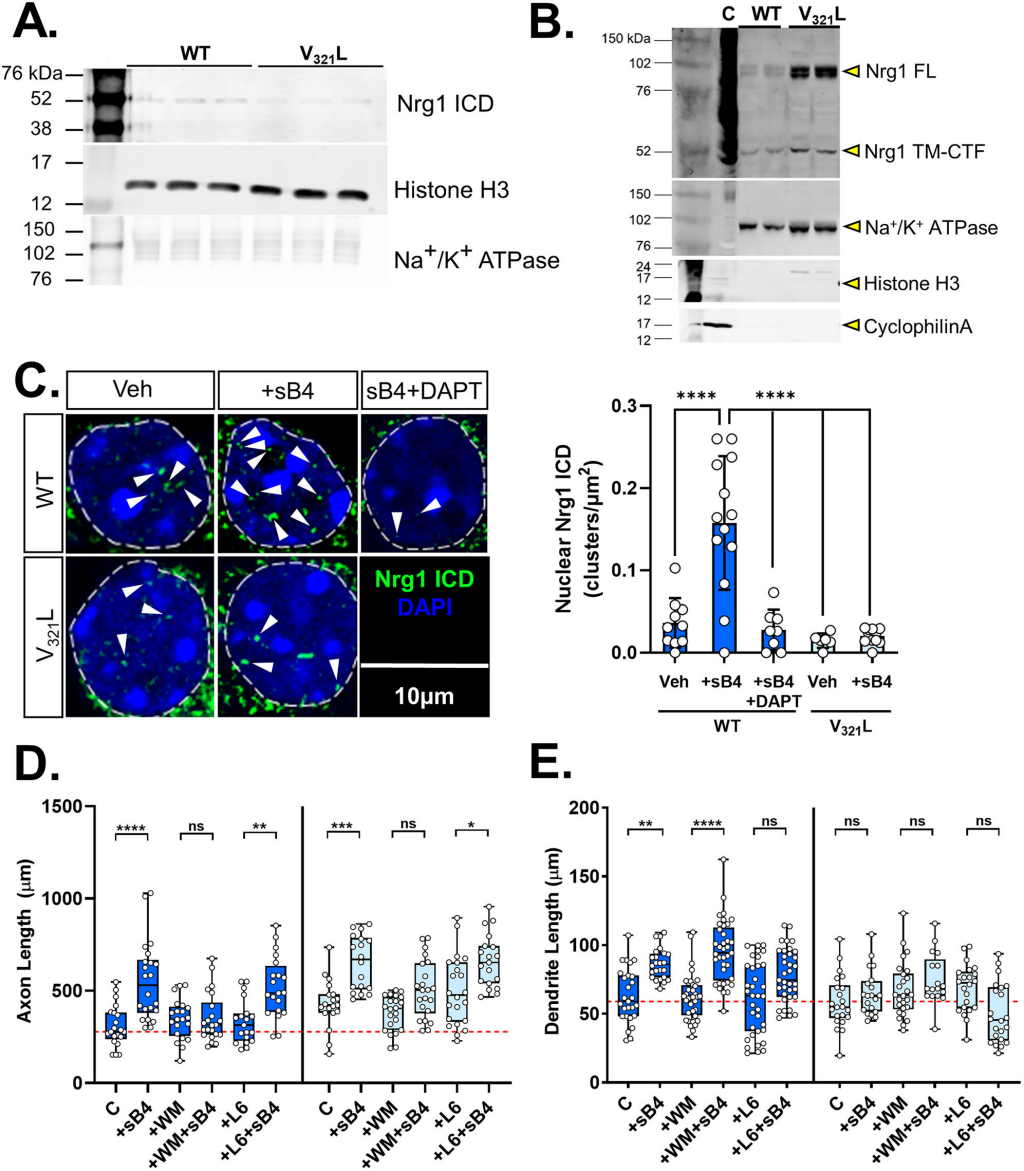
The V_321_L substitution decreases nuclear back signaling. ***A***, Immunoblot of triplicate nuclear fractions isolated from pooled cortical and hippocampal lysates. Nrg1 ICD was detected using Santa Cruz Biotechnology antibody sc-348. Histone H3 served as a nuclear loading control and Na+/K+ ATPase as a marker for the membrane fraction (*N* = 3 mice/genotype). ***B***, Immunoblot of two replicates of membrane fractions isolated from pooled cortical and hippocampal lysates. NRG1 ICD was detected using Santa Cruz Biotechnology antibody sc-348. Na+/K+ ATPase served as a marker for the membrane fraction; note the lack of the nuclear marker Histone H3 or cytoplasmic marker CyclophilinA indicating clean membrane preps. FL Nrg1 is indicated with a yellow arrowhead as “Nrg1-FL,” and the membrane-bound C-terminal fragment not cleaved by γ-secretase is indicated as “Nrg1 TM-CTF.” A positive control consisting of total lysate from N2A cells transfected with a Type III Nrg1 plasmid is shown in the lane labeled “C.” (*N* = 2 mice/genotype.) ***C***, Left, Hippocampal neurons from WT (dark blue) and V_321_L (light blue) neonatal pups (P4) were cultured for 17 d in vitro and were stimulated with either vehicle (Veh), 20 nM sERBB4 (sB4), or 20 nM sErbB4 after a 24 h pretreatment with 20 µM of the γ-secretase inhibitor DAPT (DAPT). Neurons were fixed and stained using an antibody directed against the Nrg1 ICD and counterstained with DAPI. Scale bar, 10 µm. Right, Quantification of nuclear clusters of Nrg1-ICD. Neurons from WT mice show increased nuclear ICD clusters in response to sB4 stimulation, which is counteracted by pretreatment with DAPT (DAPT). Neurons from V_321_L mice do not respond to sB4 stimulation (*N* = 6–13 neurons, 3 platings/mouse, 3 mice/genotype; one-way ANOVA *p* values corrected for multiple comparisons using Tukey’s post hoc test; WT Veh vs WT sB4, *p* < 0.0001 (****); WT sB4 vs WT sB4 + DAPT, ****p < 0.0001; WT sB4 vs V_321_L Veh, ****p < 0.0001; WT sB4 vs V_321_L B4, ****p < 0.0001). All other comparisons are statistically not statistically significant. ***D***, Cortical neurons from embryonic WT (dark blue) and V_321_L mice (light blue; E18.5) were cultured for DIV3 and were stimulated with soluble ErbB4 (sB4), PI3K inhibitor WM, γ-secretase inhibitor L-685,458 (L6), WM + B4, or L6 + B4. Neurons that underwent no drug treatments/sB4 stimulation are indicated as the control group (C). Neurons were fixed and axonal length was quantified. (Two-way ANOVA with Tukey’s post hoc correction, WT C vs WT B4, ****p = 0.0002; WT L6 vs WT L6 + B4, **p = 0.0047; V_321_L C vs V_321_L B4, ***p = 0.001; V_321_L WM vs V_321_L WM + B4, *p* = 0.1; V_321_L L6 vs V_321_L L6 + B4, *p = 0.03.) *N* = 20–37 neurons per genotype per condition. ns, not significant. ***E***, Treatment and conditions same as in ***D***. Quantification is for dendritic length. (two-way ANOVA w/ Tukey’s post hoc correction: WT C vs WT B4, **p = 0.002; WT WM vs WT WM + B4, ****p < 0.0001). *N* = 20–37 neurons per genotype per condition. ns, not significant.

The V_321_L mutation in Nrg1 impairs γ-secretase–mediated cleavage in vitro (Fleck et al., 2016). We performed subcellular fractionation to isolate nuclei and membrane fractions from cortical and hippocampal homogenates of V_321_L homozygous mutant and their WT litter- and cage mates and compared the levels of Nrg1 ICD by immunoblot analysis (Fig. 2*A*,*B*). Nuclei isolated from V_321_L animals showed lower levels of Nrg1 ICD compared with nuclei from WT counterparts (Fig. 2*A*). Concurrently, we found higher levels of FL and the membrane-bound ICD (TM-ICD) in the membrane fraction of V_321_L mice compared with that of WT mice (Fig. 2*B*). These findings indicate diminished Nrg1 nuclear back signaling in the mutant cortex and hippocampus. We next assessed stimulus-induced nuclear back signaling by the addition of soluble ectodomain of the Nrg1 receptor, Erbb4 (sB4), to cultured hippocampal neurons.

Type III Nrg1 back signaling results in the appearance of distinct clusters of the ICD in the nucleus (Bao et al., 2003). To determine whether nuclear ICD clusters were altered in neurons from V_321_L mice in response to ErbB4, we cultured dispersed hippocampal neurons from P4 WT and V_321_L mice [culture age, 17 d in vitro (DIV)]. We stimulated these cultures with soluble recombinant ectodomain of ErbB4 (sB4) and quantified nuclear ICD clusters (Fig. 2*C*, left). We found that stimulation with sB4 increased the number of nuclear ICD clusters in neurons from WT mice (Fig. 2*C*, right; WT Veh vs WT sB4 one-way ANOVA Bonferroni adj. *p* < 0.0001). This increase in nuclear ICD clusters was blocked by pretreatment with the γ-secretase inhibitor, DAPT [Fig. 2*C*, right; WT Veh vs WT (sB4) + DAPT Bonferroni adj. *p* > 0.9999]. We did not observe increased ICD clusters in neurons from V_321_L mice treated with sB4 [Fig. 2*C*, right; V_321_L Veh vs V_321_L (sB4) Bonferroni adj. *p* > 0.9999].

As mentioned before, Type III Nrg1 back signaling operates via two known mechanisms: (1) local axonal, PI3K-Akt signaling and (2) γ-secretase–dependent nuclear signaling, required for dendritic growth and complexity (Chen et al., 2010; Fazzari et al., 2014). We asked whether neurons from V_321_L mice are selectively deficient in dendrite development in response to ErbB4 stimulation. Cortical neurons from E18.5 WT and V_321_L mouse pups were cultured for DIV3. On the third day, neurons were treated with sB4 with or without pharmacological inhibition of PI3K using wortmannin (WM) or γ-secretase using L-685,458 (L6).

Neurons from WT mice showed increases in axonal and dendritic length in response to ErbB4 treatment which were blocked by WM and L6, respectively (Fig. 2*D*,*E*, dark blue boxes; axonal length, WT control vs WT sB4, *p* = 0.0002; dendritic length, WT control vs WT sB4, *p* = 0.0019). WM treatment did not block ErbB4-induced dendritic growth, and L6 treatment did not prevent sErbB4-induced axonal growth (axonal length, WT L6 vs WT L6 + sB4, *p* < 0.0001; dendritic length, WT WM vs WT WM + sB4, *p* < 0.0001). These results agree with previously published data showing that Nrg1 nuclear back signaling influences dendritic growth (Chen et al., 2010). These data also establish that stimulation of axonal growth requires PI3K signaling and that these two modes of Nrg1 back signaling are functionally independent.

Neurons from V_321_L mice showed increased axonal length in response to sErbB4 stimulation which was blocked by WM treatment and not by L6 (Fig. 2*D*, light blue boxes; axonal length, V_321_L control vs V_321_L sB4, *p* = 0.0014; V_321_L WM v. V_321_L WM + sB4, *p* = 0.1113; V_321_L L6 v. V_321_L L6 + sB4, *p* = 0.0289). On the other hand, V_321_L neurons did not show increases in dendritic length in response to sErbB4 stimulation (Fig. 2*E* dendrite length, V_321_L control v. V_321_L sB4 *p* > 0.9999). These results indicated that PI3K signaling-dependent axonal growth was intact in V_321_L mutant mice with a selective disruption of γ-secretase–dependent dendritic growth.

Thus, V_321_L mice show disruptions to regulated intramembrane proteolysis of Nrg1 and thereby to nuclear translocation of the ICD. Additionally, we demonstrate that dendritic growth in response to sErbB4 stimulation was absent in neurons from V_321_L mutant mice, whereas axonal growth in response to sErbB4 was intact.

### Developmental regulation of Nrg1 nuclear back signaling in GC cultures

**Figure 3.**
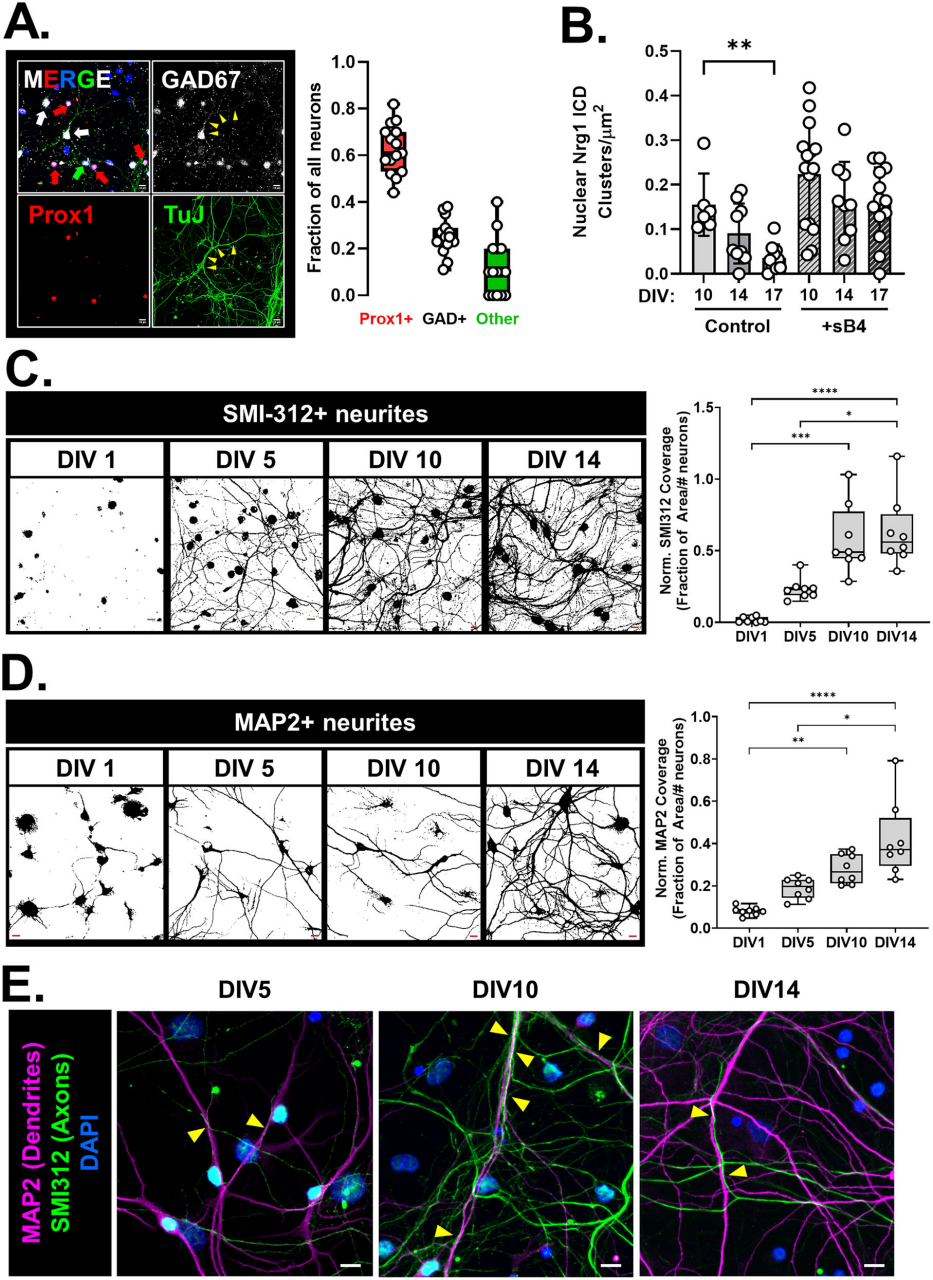
The peak of Nrg1 nuclear back signaling aligns with the peak of axon growth and precedes the peak of dendrite growth. ***A***, Characterization of neuronal types present in the P4–5 hippocampal culture. Left, Representative image from a WT P5 culture stained for GAD67 (grayscale; GABAergic neuron marker), TuJ (green; neuronal marker), Prox1 (red; GC marker), and counterstained with DAPI (blue; to label nuclei). GCs were identified as Prox1+ TuJ+ cells. GABAergic interneurons were identified as GAD67+ TuJ+ Prox1− cells, where the GAD67 staining also delineated the neurites (example indicated by yellow arrowheads). Neurons not stained by Prox1 or GAD67 were identified as other excitatory neurons likely representing pyramidal and/or mossy cells. Scale bar, 10 µm. Right, Quantification of neuronal types as proportion of all neurons. Data were pooled from cultures imaged on various days in vitro (DIV5, 10, 14). Overall, GCs make up majority of the neurons in these cultures (mean, 62.6%; SD, 10.5%). *N* = 15–16 platings from 12 to 16 mice from two litters. ***B***, Quantification of Nrg1 ICD clusters in nuclei of WT neurons from P4 to 5 hippocampal cultures at different DIVs under basal conditions (control) and after stimulation with sB4 (20 nM). *N* = 6–15 neurons/condition from platings made from three mice; Kruskal–Wallis test, **p = 0.005; KW = 10.59. (Dunn’s corrected multiple comparisons) DIV10 versus DIV17 *p* = 0.0047. Note: DIV17 group same as Figure 2*C* for comparison. ***C***, Left, Representative thresholded images of SMI-312 staining (pan-axonal neurofilament marker) from WT mice at DIV1, 5, 10, and 14. Scale bar (red), 10 µm. Right, Quantification of fraction of area covered by SMI-312 staining normalized to the number of neurons in the imaging field. In WT cultures overall axonal coverage increased until DIV10 stabilizing thereafter. Kruskal–Wallis test, *p* < 0.0001 KW = 25.79. (Dunn’s corrected multiple comparisons) DIV1 versus DIV10, ***p = 0.0003; DIV1 versus DIV14, ****p < 0.0001; DIV5 versus DIV14, *p = 0.046. All other comparisons were not significant. *N* = 8–10 platings from 6 to 8 mice from two litters for each time point. ***D***, Left, Representative thresholded images of MAP2 staining (dendrite marker at time points after DIV1) from WT mice at DIV1, 5, 10, and 14. Scale bar (red), 10 µm. Right, Quantification of fraction of area covered by MAP2 staining normalized to the number of neurons in the imaging field. Overall dendrite coverage increased till DIV14 in WT cultures. Kruskal–Wallis test, *p* < 0.0001; KW = 24.93. (Dunn’s corrected multiple comparisons) DIV1 versus DIV10, **p = 0.004; DIV1 versus DIV14, ****p < 0.0001, DIV5 versus DIV14, *p = 0.03. All other comparisons were not significant. *N* = 8–10 platings from 6 to 8 mice from two litters per genotype for each time point. ***E***, Representative images of MAP2 and SMI312 staining of WT cultures at DIV5, 10, and 14 showing increased axon–dendrite interactions at DIV10. Yellow arrowheads denote regions of axon–dendrite contacts. Scale bar, 10 µm.

In hippocampal neurons, the Type III Nrg1 protein is part of the presynaptic membrane where it is predicted to interact with ErbB4 on dendrites of GABAergic interneurons (Vullhorst et al., 2009, 2017). We noted the presence of nuclear Nrg1 ICD clusters at the baseline in our previous experiments, which increased in number following stimulation with ErbB4 (Fig. 2*C*). Thus, we next sought to examine this baseline endogenous nuclear signaling and asked whether it might correspond to a specific developmental window. We first characterized our P4 hippocampal culture preparation to ask if GABAergic interneurons were present in our culture, as a possible source of ErbB4 in the culture. Approximately 60–70% (mean, 63% ± 10% SD) of the neurons were GCs (Prox1+), whereas ∼25% (mean, 25% ± 7.5% SD) of the neurons were GABAergic (GAD67+) and the remainder ∼12% (mean, 12% ± 12% SD) were other neurons likely corresponding to glutamatergic pyramidal and mossy cells (Fig. 3*A*). Next, we assessed baseline and stimulated nuclear back signaling at DIV10, 14, and 17. We found that the baseline nuclear back signaling diminished over time in culture but remained inducible by sErbB4 treatment (Fig. 3*B*; Kruskal–Wallis test, *p* = 0.0003; KW = 23.31; DIV10 control vs DIV17 control Dunn’s adj. *p* = 0.049; DIV17 control vs DIV17 + sB4 Dunn’s adj. *p* = 0.006; DIV10 + sB4 vs DIV17 + sB4 Dunn’s adj. *p* > 0.9999). Intriguingly, at DIV10 the level of baseline nuclear ICD clusters was high, and sErbB4 treatment could not significantly enhance this signal (Dunn’s adj. *p* > 0.9999). Since Nrg1 nuclear back signaling is thought to be regulated by cell–cell interactions and in turn influences neurite growth and development, we characterized the axonal and dendritic growth over time in our GC cultures to ask whether the high basal nuclear back signaling might correspond to a particular developmental process associated with neurite growth. Axonal growth (indicated by the area covered by SMI312+ processes) increased from DIV1 to DIV10, plateauing thereafter (Fig. 3*C*; Kruskal–Wallis, *p* = 0.0001; KW = 25.79; DIV1 vs DIV10 Dunn’s adj. *p* = 0.0003; DIV1 vs DIV14 Dunn’s adj. *p* < 0.0001; DIV5 vs DIV14 Dunn’s adj. *p* = 0.0462). Qualitatively, we noted a gradual increase in axonal bundling over time. Similarly, we noted a gradual increase in dendritic growth (indicated by the area covered by MAP2+ processes) from DIV1 to DIV14 (Fig. 3*D*; Kruskal–Wallis *p* > 0.0001; KW = 24.93; DIV1 vs DIV10 Dunn’s adj. *p* = 0.0035; DIV1 vs DIV14 Dunn’s adj. *p* < 0.0001; DIV5 vs DIV14 Dunn’s adj. *p* = 0.0308). Qualitatively, we also noted an enhancement of dendritic complexity between DIV10 and 14 (Fig. 3*D*). As noted earlier, nuclear back signaling was maximal at DIV10; thus we examined interactions between axons and dendrites at DIV5, 10, and 14 to assess whether an increase in axon–dendrite contacts corresponds to the high nuclear back signaling (Fig. 3*E*). We found that at DIV5, a few thin SMI312+ processes (individual axons) were in proximity to MAP2+ processes (dendrites; Fig. 3*E*, left). This was dramatically enhanced at DIV10 where we noted a higher amount of axonal coverage, along with multiple axons “running along” dendrites (Fig. 3*E*, middle). Finally, at DIV14, we noted axonal bundling indicated by the presence of thicker SMI312+ processes, which ran along dendrites (Fig. 3*E*, right). Thus, under these culture conditions, DIV10 represents a period of dynamic axonal growth, axon–dendrite contact, and endogenous nuclear back signaling, which is followed by increased dendritic complexity. Intriguingly, this period falls squarely within the γ-secretase inhibition–sensitive window for dendrite development (see below) indicating that the nuclear ICD might regulate genes related to dendrite development.

#### γ-Secretase activity antagonistically regulates axon and dendrite development

**Figure 4.**
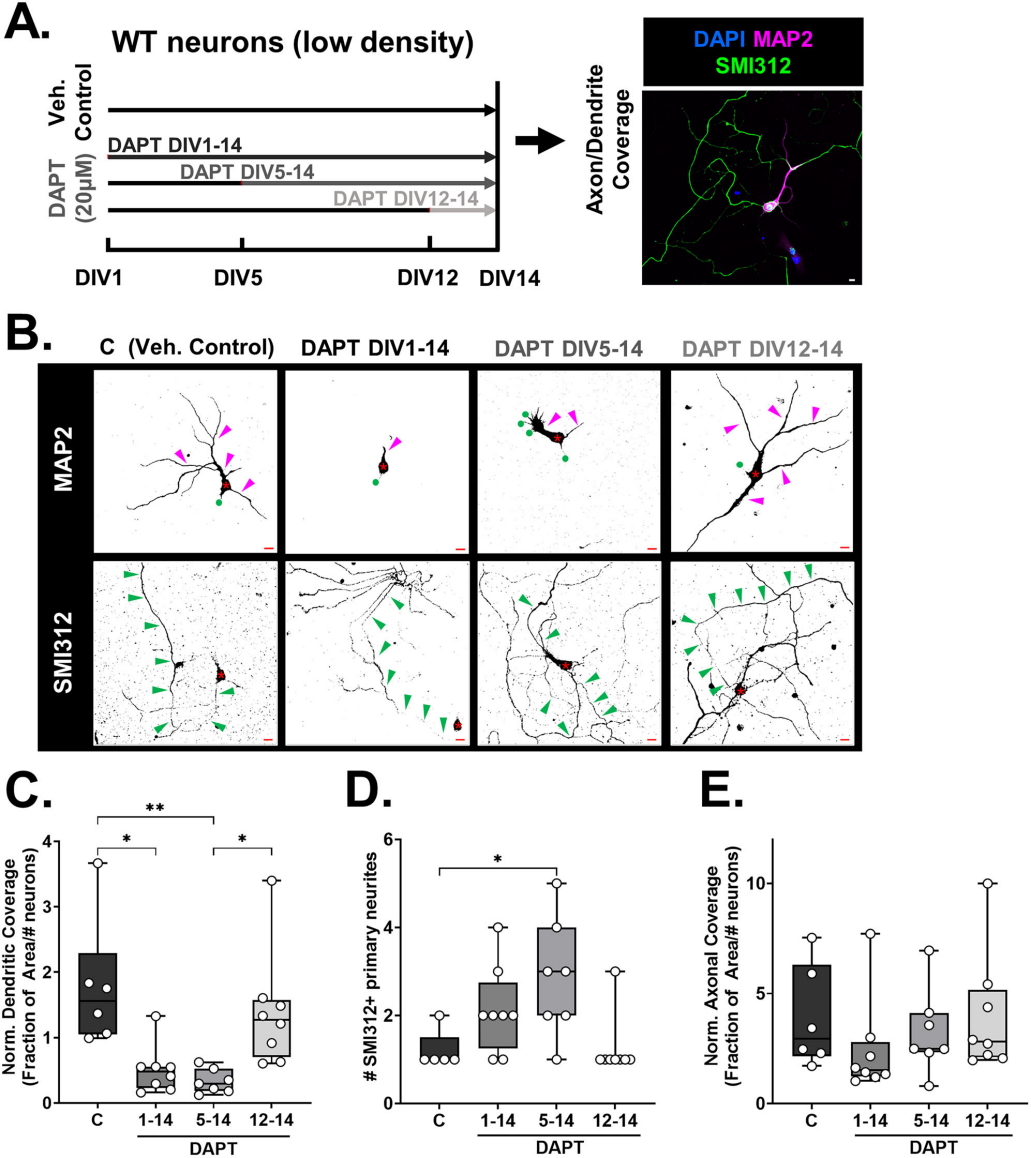
γ-Secretase regulates axon and dendrite development. ***A***, Schematic of DAPT treatment regimens that cultured neurons from WT mice (P4/5 hippocampus) were subjected to. Vehicle controls (***C***) received equal volume of DMSO diluted in culture media. The DAPT1–14 group received DAPT treatment from DIV1–14, the DAPT5–14 group was cultured for the first 5 d without DAPT but received DAPT from DIV5 onward, and the DIV12–14 group received DAPT from DIV12 onward. All platings were collected at DIV14, fixed and stained for MAP2 and SMI312. ***B***, Representative images of (top) MAP2 and (bottom) SMI312 immunostaining for each of the treatment conditions described in panel ***A***. Asterisk (*) denotes the soma of each neuron. Magenta and green arrow heads indicate MAP2+ and SMI312+ neurites, respectively. Green circles in the MAP2-stained images mark the point(s) of origin of SMI312+ neurites. The same neuron is shown in both top and bottom panels. Note that for the DAPT DIV1–14 images, the SMI312 panel was recentered to allow visualization of the axon (soma now in bottom right corner). Scale bar, 10 µm. ***C***, Quantification of fraction of area covered by MAP2 staining normalized to the number of neurons in the imaging field. Inhibition of γ-secretase prior to DIV12 significantly reduced dendritic growth. Kruskal–Wallis test, *p* = 0.0003; KW = 19.09. C versus DAPT1–14 Dunn’s corrected, *p = 0.015; C versus DAPT5–14 Dunn’s corrected, **p = 0.003; DAPT5–14 versus DAPT12–14 Dunn’s corrected, *p = 0.011. *N* = 6–8 platings from 6 to 8 mice from two litters per genotype for each time point. ***D***, Quantification of number of primary neurites immunoreactive for SMI312 after vehicle control treatment or treatment with DAPT for varying durations. Kruskal–Wallis test, *p* = 0.01; KW = 11.13. C versus DAPT5–14 Dunn’s corrected, *p = 0.04. All other comparisons were not statistically significant. *N* = 6–8 platings from 6 to 8 mice from two litters per genotype for each time point. ***E***, Quantification of fraction of area covered by SMI312 staining normalized to the number of neurons in the imaging field. Inhibition of γ-secretase had no significant effect on axonal coverage. Kruskal–Wallis test, *p* = 0.21; KW = 4.487. *N* = 6–8 platings from 6–8 mice from two litters per genotype for each time point.

The V_321_L mutation disrupts γ-secretase processing of Nrg1 thereby preventing nuclear translocation of the ICD (Fig. 2*C*). Additionally, nuclear translocation of the ICD is regulated during development in GC-enriched cultures (Fig. 3*B*). Thus, we next queried the role of γ-secretase activity in axon and dendrite development during specific developmental periods. We noted that the levels of Nrg1 ICD in the nucleus peak at or prior to DIV10 after which they decline (Fig. 3*B*). Additionally, it was after DIV10 that we noted the dramatic increase in dendritic complexity (Fig. 3*D*, left). Thus, we hypothesized that inhibiting γ-secretase during this period might influence dendrite development. We treated P4 WT hippocampal cultures with the γ-secretase inhibitor, DAPT, or vehicle (control) for varying durations of time—DIV1–14, DIV5–14, or DIV12–14 (Fig. 4*A*, left). Cultures were fixed and stained for the pan-axonal marker SMI312 and dendritic marker MAP2 on DIV14 to quantify axonal and dendritic coverage (Fig. 4*A*, right). Inhibiting γ-secretase prior to DIV12 resulted in severe dendritic growth defects (Fig. 4*B*, top; Fig. 4*C*, Kruskal–Wallis test, *p* = 0.0003; KW = 19.09; control vs DAPT1–14, *p* = 0.015; control vs DAPT5–14, *p* = 0.003; control vs DAPT12–14, *p* > 0.9999; DAPT5–14 vs DAPT12–14, *p* = 0.011). Starting γ-secretase inhibition after DIV5 was sufficient to induce the severe dendritic growth defect phenotype, indicating that there is a period of γ-secretase–dependent dendrite growth between DIV5 and 12. We also found that there was an increase in the number of primary neurites immunoreactive for the pan-axonal marker, but no significant differences in axonal coverage between the control and γ-secretase–treated groups (Fig. 4*B*, bottom; Fig. 4*D*. SMI312+ primary neurites, Kruskal–Wallis test, *p* = 0.01; KW = 11.13; C vs DAPT5–14 *p* = 0.04; Fig. 4*E*, axonal coverage, Kruskal–Wallis test, *p* = 0.21; KW = 4.487). These results indicate that γ-secretase activity between DIV5 and 12 is necessary to constrain axonal development and promote dendritic development.

#### Changes in Nrg1 V_321_L mutant DG transcriptome point to aberrant neurogenesis, cell cycle dynamics, and dendrite development

**Figure 5.**
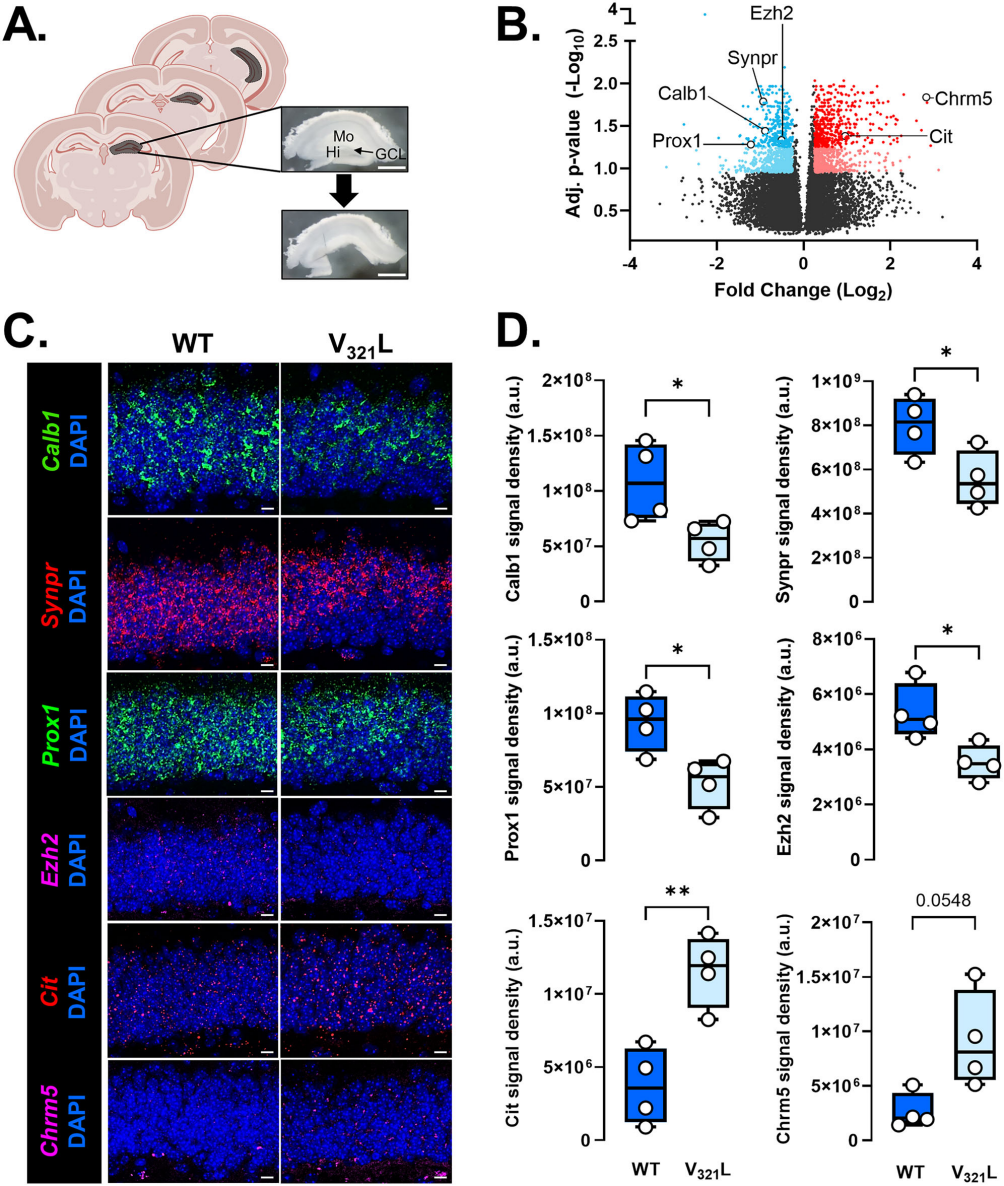
The V_321_L substitution in Nrg1 alters gene expression in the DG. ***A***, Schematic and representative image of DG dissection from hippocampal slices; Mo, molecular layer; GCL, granule cell layer; Hi, hilus. ***B***, Volcano plot for DEGs between V_321_L and WT DG. Each datapoint represents a single gene. Colored points delineate FC of at least 1.25 (blue dots, downregulated; red dots, upregulated in the mutant samples) with an adjusted *p* value of <0.1, and dark-colored points represent those with ANCOVA adjusted *p* value of <0.05. (*N* = 6 mice/genotype, 3 males/3 females). *Prox1*, *Calb1*, *Synpr*, *Ezh2*, *Cit*, and *Chrm5* were chosen for validation via RNAscope in situ hybridization. The complete list of DEGs, statistics, and pathway analyses can be found in Extended Data Tables 5-1–5-3. ***C***, Representative images of WT and V_321_L sections showing DAPI-stained DG GCL and staining for mRNA of the indicated genes in panel ***B***. ***D***, Quantification of RNAscope signal shown in panel ***A***. [From top to bottom, WT vs V_321_L Welch’s *t* test (two-tailed)] *Calb1*, *p = 0.049; *t* = 2.679; df = 4.435; *Synpr*, *p = 0.04; *t* = 2.672; df = 5.992; *Prox1*, *p = 0.02; *t* = 3.174; df = 5.882; *Ezh2*, *p = 0.03; *t* = 3.031; df = 5.034; *Cit*, **p = 0.005; *t* = 4.352; df = 5.983; *Chrm5*, *p* = 0.0548; *t* = 2.740; df = 3.808. *N* = 4 mice/genotype.

The Nrg1 ICD has strong transactivation properties (Bao et al., 2003), and V_321_L mice have impaired nuclear translocation of the ICD (Fig. 2). Thus, we predicted that impaired nuclear signaling by the Nrg1 ICD would result in transcriptomic changes. We extracted RNA from microdissected DG from WT and V_321_L mice for RNA sequencing (Fig. 5*A*). DEG analysis revealed 1,312 significantly dysregulated genes (colored dots, ANCOVA corrected *p* ≤ 0.1 and linear FC of ±1.25) between WT and V_321_L mice among which were genes specifically important for DG GC specification and function such as *Prox1*, *Calb1*, and *Synpr* (Fig. 5*B*; Extended Data Table 5-1). To identify possible functional alterations that might result from transcriptional dysregulation in V_321_L mice, we used IPA to find cellular processes that might be affected by the significantly dysregulated genes. Several processes along the neurodevelopmental trajectory were predicted to be disrupted in the V_321_L DG, including cell proliferation, differentiation, and dendrite development (Extended Data Table 5-2). Disease annotations in IPA indicated that dysregulated genes in the V_321_L DG were enriched in genes associated with SCZ susceptibility, indicating that the V_321_L mutation alters the transcriptional landscape in ways that have been associated with the genetic architecture of SCZ (Extended Data Table 5-3). We also found that dysregulated genes in V_321_L mice were enriched in cancer-associated genes, consistent with predicted changes in regulation of cell proliferation (Extended Data Table 5-3).

10.1523/JNEUROSCI.0063-24.2024.t5-1 Table 5-1 **Differentially expressed genes in V_321_L mutant DG compared to WT DG.** Differential gene expression between V_321_L and WT DG adjusted for biological sex and batch effects along with various statistical measures from ANCOVA. Download Table 5-1, XLSX file.

10.1523/JNEUROSCI.0063-24.2024.t5-2 Table 5-2 **Ingenuity pathway analysis (IPA) for functional and pathway annotations. (Top)** Functional annotations of differentially expressed genes in the V_321_L DG compared to WT DG. **(Bottom)** Canonical pathways implicated by the differentially expressed genes in the V_321_L DG. P-values and predicted activation state and z-scores. Download Table 5-2, XLSX file.

10.1523/JNEUROSCI.0063-24.2024.t5-3 Table 5-3 **Ingenuity pathway analysis (IPA) for disease annotations.** Disease annotations of differentially expressed genes in the V_321_L DG compared to WT DG. Download Table 5-3, XLSX file.

To validate the results from our RNA-Seq experiment, we performed FISH using the RNAscope assay on WT and V_321_L DG brain sections and quantified the expression of a few top differentially expressed genes (DEGs; indicated in Fig. 5*B*) in the GCL of the dorsal DG—*Prox1*, *Calb1*, *Synpr*, *Ezh2*, *Cit*, and *Chrm5* (Fig. 5*C*). In line with our RNA-Seq data, expression of *Prox1*, *Calb1*, *Synpr*, and *Ezh2* was significantly lower in the V_321_L DG compared with WT (Fig. 5*D*, *Prox1*, WT vs V_321_L, *p* = 0.02; *t* = 3.174; df = 5.882; *Calb1*, WT vs V_321_L, *p* = 0.05; *t* = 2.679; df = 4.435; *Synpr*, WT vs V_321_L, *p* = 0.04; *t* = 2.672; df = 5.992; *Ezh2*, WT vs V_321_L, *p* = 0.03; *t* = 3.031; df = 5.034) and expression of *Cit* and *Chrm5* was higher (Fig. 5*D*, *Cit*, WT vs V_321_L, *p* = 0.005; *t* = 4.352; df = 5.983; *Chrm5*, WT vs V_321_L, *p* = 0.05; *t* = 2.740; df = 3.808). We did not find any significant differences in the DAPI + GCL area between WT and V_321_L mice indicating no overt difference in numbers of GCs between the genotypes (*p* = 0.3429; Mann–Whitney rank sum test, *U* = 4).

#### Shared TR mechanisms among DEGs in the V_321_L DG implicate dysregulation of the polycomb repressor complex 2 (PRC2)

We next asked whether the DEGs shared TR mechanisms using ENCODE and ChEA databases (Lachmann et al., 2010; Chen et al., 2013; Kuleshov et al., 2016; Xie et al., 2021). The 664 significantly upregulated genes in V_321_L mice were enriched for genes predicted to be regulated by members of the PRC2 (SUZ12, EZH2), RE1-silencing transcription factor (REST), and the CTCF–cohesin complex (CTCF, RAD21, SMC3) involved in chromatin looping (Extended Data Table 5-4). We grouped these regulators into two higher-order groups: (1) PRC2 + REST and (2) CTCF–cohesin. We found that 126 of the 664 upregulated genes were targets for regulation by the PRC2, and 81 of the 664 upregulated genes were targets of REST; 28 of these 81 REST-target genes (∼35%) were also targets of the PRC2. Gene ontology analyses for the PRC2 target genes revealed enrichment of genes predicted to be involved in synapse assembly, synaptic transmission, and neuronal differentiation (Extended Data Table 5-5). Intriguingly, we found that EZH2 and EED (catalytic subunits of PRC2) were downregulated in the RNA-Seq comparison between WT and V_321_L DG (Extended Data Table 5-1; Fig. 5*D*). The CTCF–cohesin-regulated genes were enriched for functions such as axon guidance, cell migration, synapse assembly, and protein phosphorylation.

10.1523/JNEUROSCI.0063-24.2024.t5-4 Table 5-4 **Transcriptional Regulators (TRs) of DEGs in the V_321_L DG. (Top)** Predicted TRs for genes upregulated in the V_321_L DG. **(Bottom)** Predicted TRs for genes downregulated in the V_321_L DG. Download Table 5-4, XLSX file.

10.1523/JNEUROSCI.0063-24.2024.t5-5 Table 5-5 **Gene Ontology (GO) analysis of biological processes served by DEGs predicted to be regulated by the polycomb repressor complex and E2F4. (Top)** GO biological processes regulated by DEGs in V_321_L DG predicted to be regulated by components of the PRC2. **(Bottom)** GO biological processes regulated by DEGs in V_321_L DG predicted to be regulated by E2F4. Download Table 5-5, XLSX file.

The 647 downregulated genes showed strongest enrichment of genes predicted to be regulated by the transcription factor E2F4 (odds ratio, 3), upstream binding transcription factor, Sin3 Transcription Regulator Family Member A (SIN3A), and Myc-associated factor X (MAX; Extended Data Table 5-4). The E2F4 target genes were found to be involved in mitotic spindle organization, microtubule organization, RNA splicing, and DNA replication (Extended Data Table 5-5). Overall, the downregulated genes were found to be involved in positive regulation of mitosis.

The observed changes in gene expression strongly implicate the Nrg1 ICD as a regulator of E2F4 and PRC2 function during a neurodevelopmental program. Based on these data, we predicted that there would be altered proliferation and neural differentiation within the V_321_L mutant neurogenic niche.

#### DEG in the V_321_L DG can be largely accounted for by a loss of nuclear back signaling

**Figure 6.**
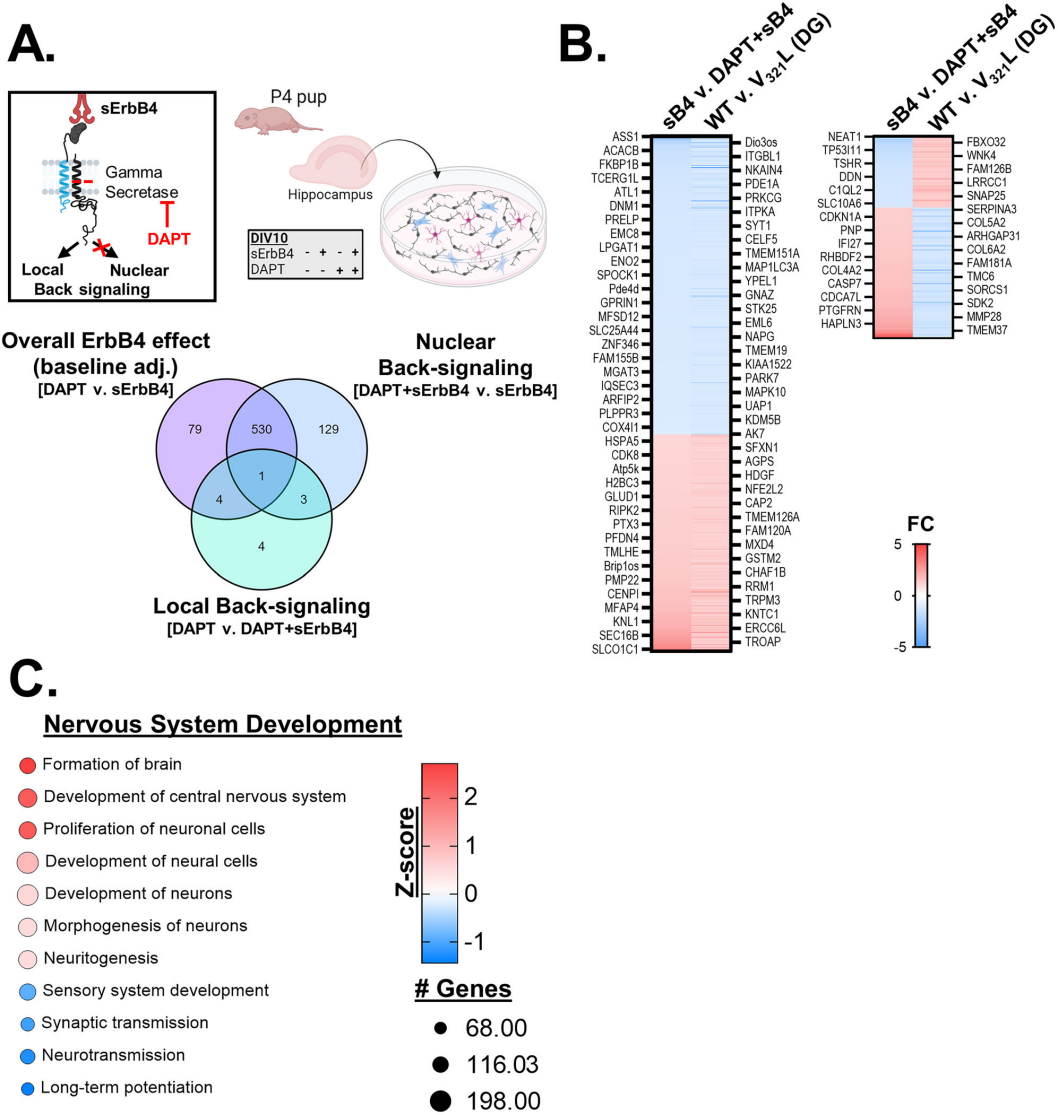
DEG in the V_321_L DG can be largely accounted for by a loss of nuclear back signaling. ***A***, Top, Schematic of the experimental paradigm. P4–5 hippocampal cultures were maintained for 8–9 d after which they were treated with DMSO (vehicle control) or 20 µM DAPT overnight. On DIV9–10, cultures were stimulated with 20 nM soluble ErbB4 (sErbB4) or equal volume 1× PBS in culture media (vehicle control) for 4 h prior to RNA extraction. The four treatment conditions are shown: vehicle alone, sErbB4 alone, DAPT alone, DAPT + sErbB4. *N* = 3 platings from 6 to 8 mice from three litters per genotype for each condition. Bottom, Venn diagram showing overlap in the DEGs (FC >1.25 in either direction; post hoc corrected, *p* < 0.05). Local back signaling did not result in significant changes in gene expression. High overlap in the baseline-adjusted ErbB4 effect and the nuclear back signaling effect indicates a high level of baseline back signaling at DIV9–10 consistent with data shown in Figure 3*B*. Also see Extended Data Tables 6-1 and 6-2. ***B***, Left, Heatmaps showing changes in gene expression in response to sErbB4 treatment compared with expression of the same genes in the WT DG tissue relative to V_321_L DG tissue for genes whose expression is changed in the same direction in vitro and in vivo. All genes detected in both datasets irrespective of the degree of FC are displayed. Each row represents a gene, and every 10th row is labeled with the gene name on either side of the heatmap. The genes are ordered from most downregulated (blue) to most upregulated (red) genes from the in vitro experiment. Color of the heatmap represents the FC in gene expression given by the legend displayed alongside. (Right) Heatmaps showing changes in gene expression in response to sErbB4 treatment compared with expression of the same genes in WT DG tissue relative to V_321_L DG tissue for genes whose expression is oppositely regulated in vitro compared with in vivo. Each row represents a gene, and every 10th row is labeled with the gene name on either side of the heatmap. The genes are ordered from most downregulated (blue) to most upregulated (red) genes from the in vitro experiment. Color of the heatmap represents the FC in gene expression given by the legend displayed alongside. ***C***, DEGs were subjected to pathway enrichment analysis using IPA, which identified significantly enriched pathways represented by the DEGs as well as a *z*-score of direction in which these pathways are predicted to be altered based on the directions of changes in expression of the genes comprising each pathway. Each pathway is indicated by a circle with the size of the circle indicating the proportion of genes comprising that pathway in the IPA database that are represented in the DEG set. The color of the circles is according to the heatmaps displayed alongside indicating the adjusted *p* values. Red pathways are predicted to become upregulated, and blue pathways are predicted to become downregulated based on the aggregate gene expression changes effected by Nrg1 nuclear back signaling.

Bulk RNA-Seq of the tissue offered us a snapshot into steady-state differences in gene expression between WT and V_321_L DG. To determine which of these gene expression differences, if any, might reflect a direct effect of altered nuclear back signaling, we measured the effect of stimulating nuclear back signaling on gene expression in cultured P4 hippocampal neurons at DIV10 (Fig. 6*A*, top). We pretreated the cultures with the γ-secretase inhibitor (DAPT) or vehicle on DIV9 for 24 h, following which we stimulated the cultures with sErbB4 or vehicle. RNA was collected 4 h after ErbB4 stimulation and sequenced. We compared the following conditions to identify Nrg1 nuclear back signaling-regulated genes: (1) DAPT versus sErbB4 (overall back signaling effect adjusted for baseline signaling), (2) DAPT versus DAPT + sErbB4 (local back signaling effect), and (3) DAPT + sErbB4 versus sErbB4 (nuclear back signaling effect adjusted for local back signaling; Fig. 6*A*, bottom). We found that local back signaling did not result in significant gene expression changes at this time point (12 DEGs). Nrg1 nuclear back signaling resulted in 663 DEGs (Extended Data Table 6-1; Fig. 6*A*, bottom).

10.1523/JNEUROSCI.0063-24.2024.t6-1 Table 6-1 **Differentially expressed genes in response to nuclear back signaling (DB vs. B) in P4-5 cultured hippocampal neurons.** Differential gene expression between DAPT + sErbB4 vs. sErbB4 conditions. Download Table 6-1, XLSX file.

10.1523/JNEUROSCI.0063-24.2024.t6-2 Table 6-2 **Ingenuity pathway analysis (IPA) for functional and pathway annotations for nuclear back signaling regulated genes. (Top)** Pathway annotations. **(Middle)** Functional annotations **(Bottom)** Disease annotations. P-values and predicted activation state and z-scores. Download Table 6-2, XLSX file.

We next compared the change in the expression of individual genes due to the V_321_L mutation in the DG and the change in expression of the same genes from acutely stimulated nuclear back signaling (Fig. 6*B*). The bulk of the genes found to be differentially expressed in the V_321_L DG were also found to be acutely regulated by nuclear back signaling, i.e., genes upregulated by stimulating nuclear back signaling in vitro showed higher expression in WT DG compared with the V_321_L DG and vice versa (Fig. 6*B*). A smaller number of genes showed the opposite effect, i.e., genes upregulated by stimulating nuclear back signaling were found to be lower in expression in the WT DG compared with that in V_321_L DG indicating potential compensatory mechanisms and/or other regulators of these genes (Fig. 6*B*). Gene ontology analyses of the nuclear back signaling-regulated genes indicated significant enrichment of genes involved in proliferation of neuronal cells and regulation of neuronal morphogenesis, consistent with predictions from the RNA-Seq of the DG tissue from V_321_L mice (Fig. 6*C*).

### Abnormal progenitor pool maintenance and differentiation in the V_321_L mutant DG

**Figure 7.**
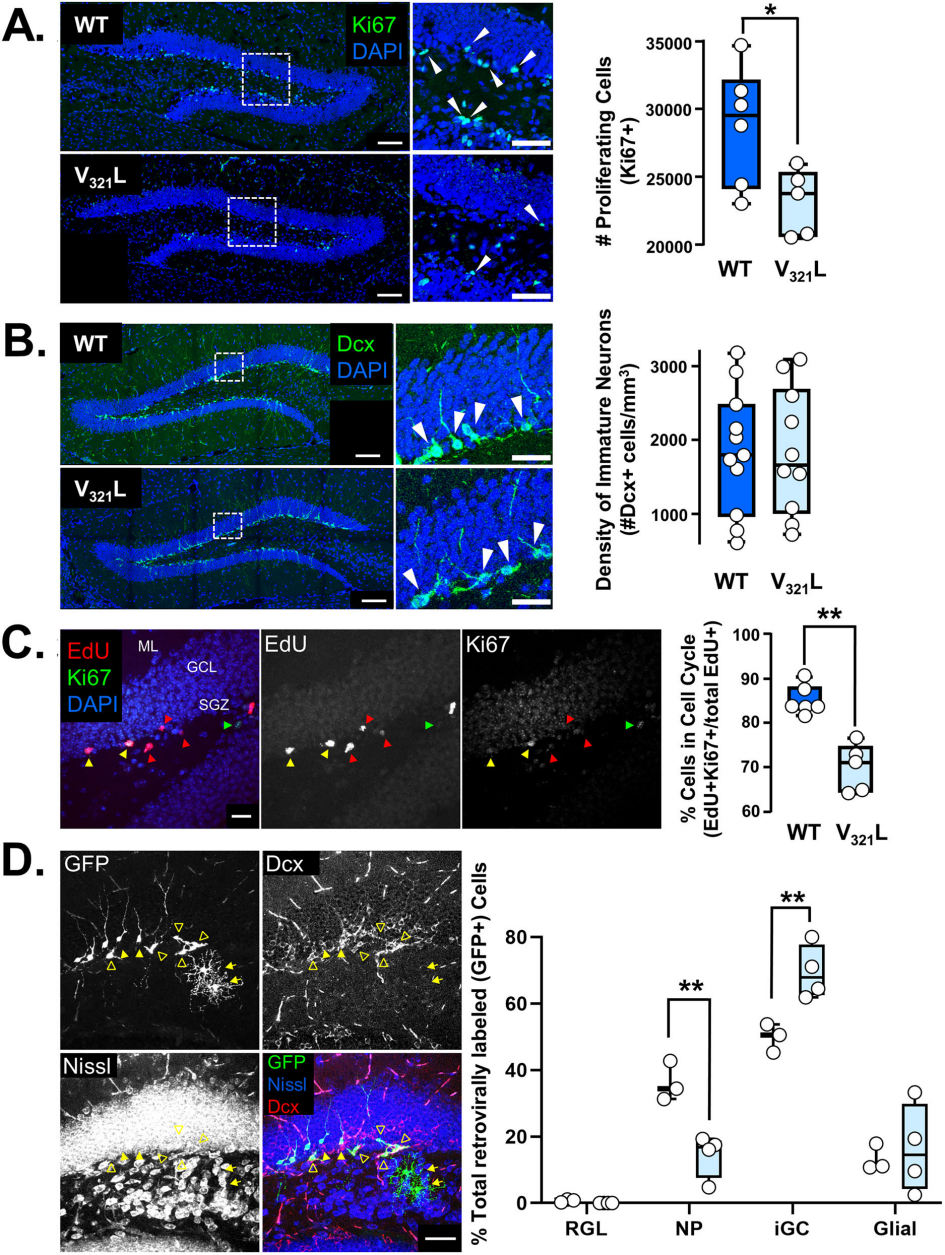
V_321_L DG shows reduced stem cell proliferation, increased cell cycle exit and commitment to neuronal fate. ***A***, Left, immunohistochemistry of Ki67 (green), a marker for proliferation, in the DG of P22 WT and V_321_L littermates and cage mates, merged with DAPI (blue) counterstain; scale bar, 100 µm. Inset shows a higher-magnification image with arrowheads indicating Ki67+ proliferating cells in the subgranular zone of the DG; scale bar, 50 µm. Right, Stereological quantification of Ki67+ populations in the DG of P21 WT and V_321_L (WT *N* = 6 mice; V_321_L *N* = 5 mice; *p = 0.03; Welch’s *t* test, *t* = 2.697; df = 8.050). ***B***, Left, Doublecortin (Dcx) immunoreactivity (green) merged with DAPI (blue) counterstain in DG of WT and V_321_L mice. Inset shows higher-magnification image of the region delineated in the white box; arrowheads point to Dcx+ cells in the DG. Right, Quantification of Dcx+ cell numbers from confocal images of subsampled, coronal DG sections. Confocal images were acquired in 3 µm *z*-steps from 20 µm coronal sections, and 4–10 images were quantified for each animal (P60–180 WT *N* = 11 mice; V_321_L = 10 mice; *p* = 0.9865; Welch’s *t* test, *t* = 0.017; df = 18.68). ***C***, Left, Image from a WT DG showing NPs in S phase of the cell cycle labeled with EdU by intraperitoneal injection (50 µg EdU/g body weight) in ∼P90 animals. Twenty-four hours postinjection, samples were harvested and processed to detect Ki67 immunoreactivity. EdU and Ki67 coimmunoreactivity indicates that cells remained in the cell cycle 24 h postinjection (yellow arrowheads). EdU immunoreactivity without Ki67 expression indicates that cells exited the cell cycle (red arrowheads). Ki67 immunoreactivity alone indicates cells that were not in S phase at the time of injection but were in the cell cycle at the time of tissue harvest. Right, Quantification of cell cycle reentry by computing the proportion of cells that remained in the cell cycle (EdU+ Ki67+) out of all cells in S phase of the cell cycle at the time of labeling (EdU+ cells). Epifluorescent images were acquired in 1 µm *z*-steps from 20 µm coronal sections, and 12–14 sections were quantified for each animal (WT *N* = 6 mice; V_321_L *N* = 5 mice, **p = 0.004, Mann–Whitney rank sum test, *U* = 0). ***D***, Left, Image from a WT DG showing neural stem cells infected with a replication-deficient GFP–expressing retrovirus via stereotaxic injection, followed by 14 d of incubation to label NPs and their progeny. The identities of GFP+ cells were determined by a combination of morphology, Dcx immunoreactivity, and Nissl staining. NPs were identified by the lack of radially oriented processes with or without Dcx immunoreactivity (open arrowheads). iGCs were identified by their radially oriented neurites, Dcx+ and Nissl+ staining (filled, yellow arrowheads). Glial cells were identified by their star-like morphology and lack of Dcx immunoreactivity and Nissl staining (yellow arrows). Right, Fate specification was quantified as proportion of GFP+ cells that remained radial glial-like stem cells, NPs, or committed to neuronal or glial fates. Five to fourteen 50 µm sections were quantified from each animal, depending on infection efficiency (WT *N* = 3 mice; V_321_L *N* = 4 mice; two-way ANOVA genotype × cell fate, *p* = 0.0003; *F*_(3, 20)_ = 9.864. NP, WT vs V_321_L Bonferroni-corrected, **p = 0.003. iGC, WT vs V_321_L Bonferroni’s-corrected, **p = 0.007. RGL and glial comparisons between genotypes were not significant; *p* > 0.9999).

We next quantified the effect of the V_321_L mutation on adult neurogenesis in the DG. Stereological analysis revealed a statistically significant decrease in the proliferative population (Ki67+) in V_321_L animals compared with that in WT (Fig. 7*A*; *p* = 0.03, Welch’s *t* test), whereas newborn neuron (Dcx+) numbers were comparable between V_321_L and WT animals (Fig. 7*B*; *p* = 0.99, Welch’s *t* test). Thus, while proliferation of stem cells was stunted in the V_321_L DG, this impairment did not result in an appreciable difference in the number of newborn neurons produced.

The discrepancy between decreased proliferation and unchanged neuronal production in the V_321_L DG could be explained by disruptions to the balance between self-renewal maintenance and postmitotic neuronal differentiation, both of which are processes predicted to be disrupted by the V_321_L mutation based on the transcriptomic data (Extended Data Table 5-2). Therefore, we asked whether cell cycle dynamics and/or fate commitment by neural stem cells were abnormal in the V_321_L DG.

To measure the rate of cell cycle reentry, we first labeled the cells in S phase of the cell cycle by systemic EdU injection. Twenty-four hours after EdU injection, we collected brain sections and stained them for Ki67 expression. We calculated the overall rate of cell cycle reentry as the proportion of total EdU+ cells that were also Ki67+. We observed a statistically significant decrease in cell cycle reentry in the V_321_L DG compared with that in WT DG (Fig. 7*C*; *p* = 0.0043; Mann–Whitney rank sum test). Therefore, the self-renewing pool of NPs was depleted faster, generating more postmitotic cells in the V_321_L mutant DG.

Given the higher rate of cell cycle exit in V_321_L DG, we asked if fate programming was affected. V_321_L and WT DGs were injected with a replication-deficient retrovirus encoding GFP, which only stably infects cell during mitosis. After 14 d, brains were collected for identification of GFP+ progeny. The identity of GFP+ cells were determined by a combination of morphology, Dcx immunoreactivity, and Nissl staining. NPs were identified by short, tangential processes and the lack of radially oriented processes, with or without Dcx immunoreactivity (Fig. 7*D*, open arrowheads). iGCs were identified by their radially oriented neurites and Dcx+ Nissl+ staining (Fig. 7*D*, closed arrowheads). Glia (astrocytes/oligodendrocytes) were identified by their star-like morphology, extensive branch ramifications, and lack of Dcx immunoreactivity and Nissl staining (Fig. 7*D*, yellow arrows).

The results revealed a decrease in the proportion of NPs in the V_321_L DG compared with that in WT DG (Fig. 7*D*; two-way ANOVA genotype × cell fate, *p* = 0.0003; *F*_(3,20)_ = 9.864; NP, WT vs V_321_L Bonferroni-corrected, *p* = 0.0026), consistent with the observed depletion of the proliferative pool (Fig. 7*A*) and increased rate of cell cycle exit (Fig. 7*C*). Interestingly, fate commitment in the V_321_L DG was disproportionately biased toward the neuronal fate program compared with glial fate (Fig. 7*D*; iGC, WT vs V_321_L Bonferroni-corrected, *p* = 0.0069). In fact, the proportion of glia generated by the V_321_L neural stem cells was not significantly different between V_321_L and WT DG (glia, WT vs V_321_L *p* > 0.9999).

Thus, we found depletion of the proliferative neural stem cell pool, increased cell cycle exit, and disproportionate production of neurons in the V_321_L DG in agreement with predictions from our transcriptomic analyses (Fig. 5). These findings point to an important role for Nrg1 nuclear back signaling for neural stem cell maintenance and fate commitment.

#### The Nrg1 V_321_L mutation compromises maturation of GCs in the DG

Nrg1 nuclear back signaling is important for dendrite arborization and spine production (Chen et al., 2010; Fazzari et al., 2014). V_321_L cortical neurons showed loss of ErbB4-induced dendrite growth (Fig. 2*E*), inhibition of γ-secretase resulted in a dramatic reduction in dendrite growth (Fig. 3*C*), and nuclear back signaling-regulated genes were predicted to enhance the development of neurites (Fig. 6*C*). Our RNA-Seq analysis of the V_321_L DG also showed dysregulation of genes involved in dendrite development (Extended Data Table 5-2). Therefore, we first examined the dendrite morphology of newborn dentate GCs.

**Figure 8.**
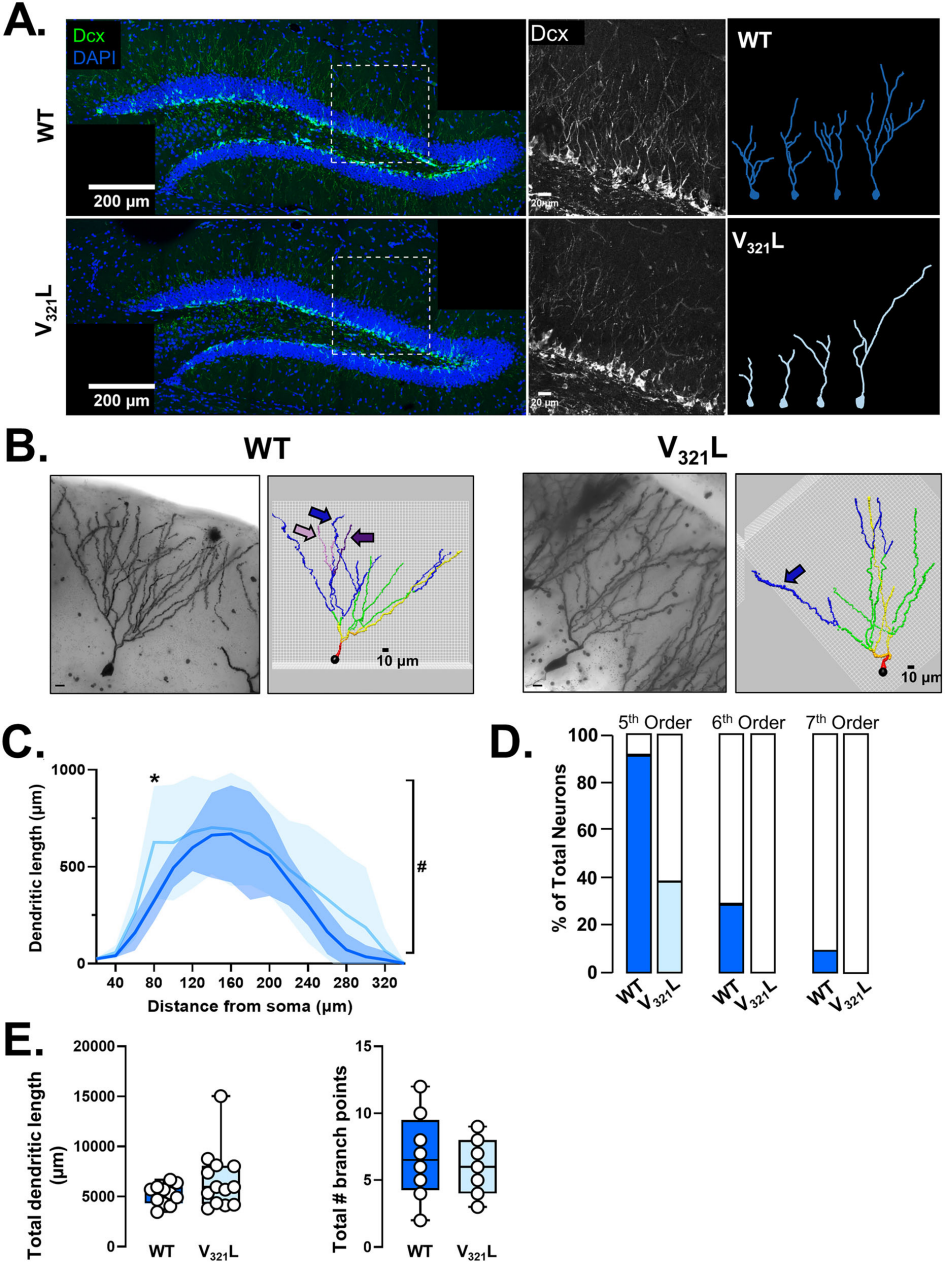
GCs produced in the V_321_L DG display aberrant dendritic arborization. ***A***, Left, Doublecortin (Dcx, green) immunohistochemistry in the P72 DG along with DAPI counterstain (blue), with high magnification of the dotted white boxes in the middle. Right, Representative tracings of Dcx+ cells in WT and V_321_L DG. ***B***, Representative images and tracings of Golgi-impregnated DG GCs from ∼P60 WT and V_321_L. Neurites are color coded by branch order (1°, red; 2°, orange; 3°, yellow; 4°, green; 5°, blue; 6°, purple; 7°, pink). A representative neuron is shown for each genotype. Arrows following the color code outlined above highlight the absence of higher-order branching in V_321_L GCs. ***C***, Length of dendritic segments within each zone were summed. Sholl analysis reveals difference in distribution of dendritic lengths as a function of distance from the soma in V_321_L mice. Shaded region around the line represents standard deviation. Data are compiled from 11 neurons from five WT (dark blue) animals and 13 neurons from five V_321_L (light blue) at ∼P60. There was a significant difference in the sholl analysis profile between WT and V_321_L neurons (mixed effect model two-way RM ANOVA genotype × distance from soma, ^#^*p* = 0.03; *F*_(16,352)_ = 1.763; WT vs V_321_L at 80 µm away from soma FDR-corrected, *q = 0.05). ***D***, Proportion of neurons in which fifth-, sixth-, and seventh-order branching are present. V_321_L GCs had fewer branches at the higher orders of branching (5th order, *p* = 0.01; 6th order, *p* = 0.08; 7th order, *p* = 0.46; Fisher’s exact test). [WT (dark blue) *N* = 11 neurons/5 mice, V_321_L (light blue) *N* = 13 neurons/5 mice]. ***E***, Left, Total dendritic length of Golgi-impregnated GCs quantified using NeuronStudio. Total dendritic length trends greater in V_321_L mice, though not statistically significant (*p* = 0.25, Wilcoxon rank sum test). Right, Total number of branch points trends lower in V_321_L DGs compared with WT counterparts, although the difference is not statistically significant (*p* = 0.45, Welch’s *t* test; WT *N* = 11 neurons/5 mice; V_321_L, *N* = 13 neurons/5 mice).

Qualitative observations from Dcx immunohistochemistry revealed decreased dendritic complexity of adult-born immature neurons in V_321_L DG (Fig. 8*A*). To systematically quantify mature GC dendritic arbors, we used Golgi impregnation to visualize neuronal morphology and only analyzed dendrites of neurons with typical GC morphologies (see Materials and Methods for details on criteria). Figure 8*B* shows representative tracings of GCs from each genotype. Sholl analysis revealed significant increases in V_321_L dendritic length proximal to the soma [Fig. 8*C*; at 80 µm away from soma Bonferroni-corrected, *p* = 0.052; two-way RM ANOVA with a significant (genotype × distance from soma) interaction, *p* = 0.035].

Dendrite complexity appeared to be compromised in GCs sampled from V_321_L DGs. In WT neurons, most of the dendritic length was distributed in fourth and fifth order, whereas in V_321_L GCs, the peak of dendritic length was found in fourth-order branches with less length distributed in fifth-order branches (data not shown). In fact, there was a statistically significant decrease in the proportion of GCs that possessed fifth-order branching in V_321_L GCs, and while a small percentage of WT GCs possessed sixth- and seventh-order branches, these high-order branches were not observed in V_321_L GCs (Fig. 8*D*, fifth order, *p* = 0.0131; sixth order, *p* = 0.0815; seventh order, *p* = 0.458; Fisher’s exact test). Total dendritic length and number of branch points were not significantly altered in the V_321_L DG (Fig. 8*E*; total length, *p* = 0.2518; Mann–Whitney rank sum test, *U* = 51; branch points, *p* = 0.6035; Welch’s *t* test, *t* = 0.5331; df = 12.23).

Altogether, examination of dentate GCs revealed alterations in the dendrites in both immature and mature V_321_L GCs, especially in distal dendrites. Thus, diminished Nrg1 nuclear back signaling results in persistent and cumulative effects on dendrite growth and complexity across development.

#### Cell autonomous rescue of dendritic growth by the Nrg1 ICD

**Figure 9.**
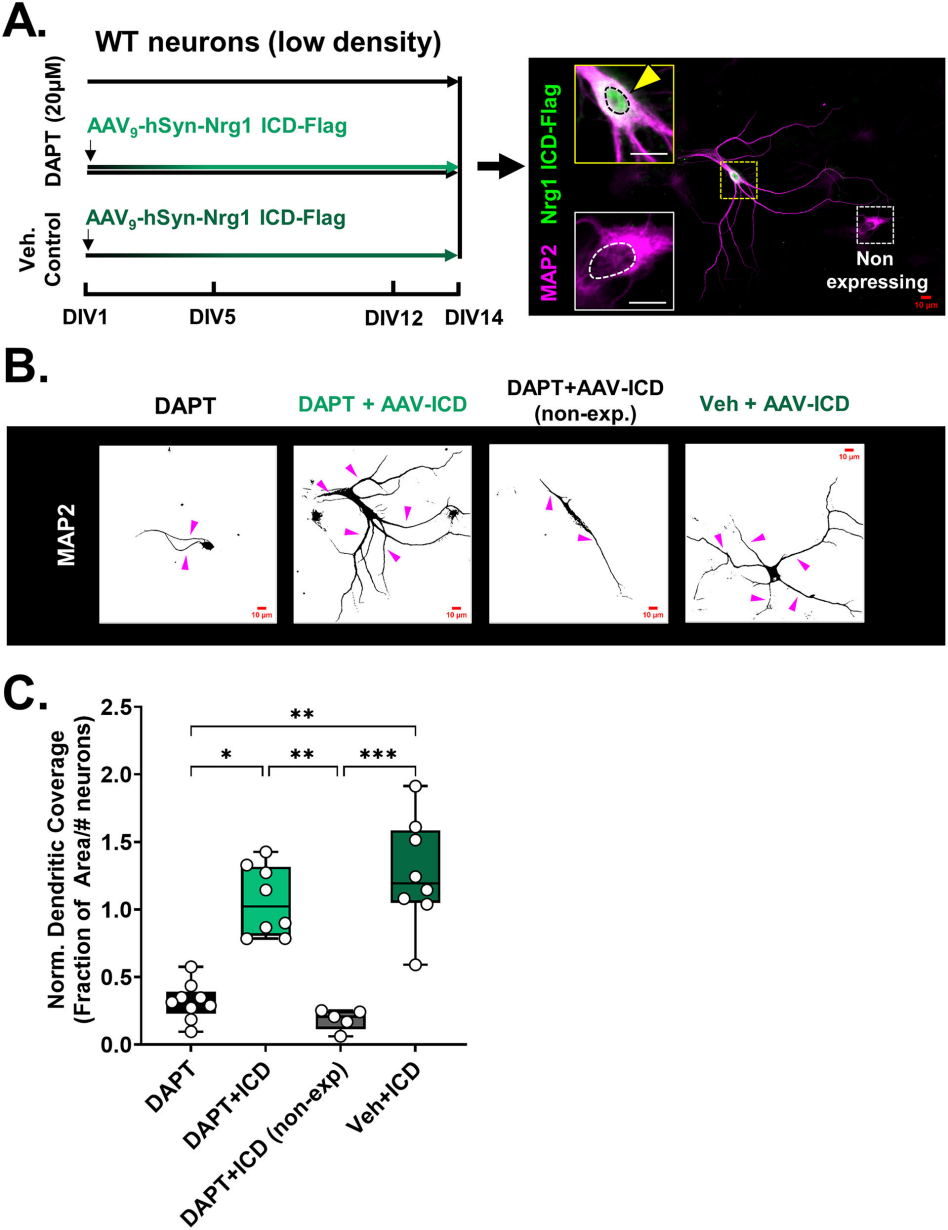
γ-Secretase–dependent dendrite growth requires the nuclear Nrg1 ICD. ***A***, Left, Schematic of the various treatment regimens. Vehicle controls received equal volumes of DMSO diluted in culture media. DAPT treatment was started from DIV1 and continued until DIV14. One of the DAPT treatment groups and the vehicle control group, on DIV1, were also incubated with AAV9 particles to deliver an expression construct for the Nrg1 ICD (flag-tagged) under the control of the human synapsin (hSyn) promoter. All platings were collected at DIV14, fixed and stained for MAP2 and Flag. Right, Representative image of a culture treated with DAPT from DIV1 to 14 and infected with AAV_9_-hSyn-Nrg1ICD-Flag, stained for MAP2 and Flag on DIV14. The image shows two neurons in the same plating: one that is expressing the ICD and another that is nonexpressing. Insets show each of these cells at higher magnification, and the dotted line delineates the nucleus. The yellow arrowhead indicates Nrg1 ICD expression. Scale bar, 10 µm. ***B***, Representative images of MAP2 staining at DIV14 for each of the treatment conditions described in panel ***A***. Magenta arrowheads indicate MAP2+ neurites. Scale bar, 10 µm. ***C***, Disrupted dendrite outgrowth by γ-secretase inhibition was rescued by expression of the nuclear Nrg1 ICD. Quantification of fraction of area covered by MAP2 staining normalized to the number of neurons in the imaging field. Inhibition of γ-secretase significantly reduced dendritic growth, which was rescued by reexpression of the nuclear Nrg1 ICD. Kruskal–Wallis test, *p* = <0.0001; KW = 22.94. DAPT versus DAPT + ICD, *p = 0.03; DAPT versus Veh + ICD, **p = 0.004; DAPT + ICD versus DAPT + ICD (no exp), **p = 0.0045; DAPT + ICD (no exp) versus Veh + ICD, ***p = 0.0007. *N* = 8–10 platings from 6 to 8 mice from two litters per genotype for each condition.

Nrg1 nuclear back signaling requires γ-secretase activity (Fig. 2*C*), and γ-secretase activity is necessary for proper dendrite development in GC-enriched hippocampal cultures (Fig. 3*C*). Finally, dysregulated gene expression in the V_321_L DG are enriched for genes involved in dendrite morphogenesis, and mutant mice show altered GC dendritic morphology (Extended Data Table 5-2, Fig. 8). Thus, we hypothesized that the γ-secretase–dependent signal required for dendritic growth might be the nuclear Nrg1 ICD. To test this hypothesis, we cultured P4 hippocampal neurons in the presence of DAPT from DIV1–14 as described earlier. To the one group of DAPT-treated neurons and one group of vehicle-treated neurons, we added AAV_9_ particles to deliver an expression construct for the Nrg1 ICD-Flag (AAV-ICD; Fig. 9*A*, left). Neurons were cultured for 14 d after which dendritic growth was assessed via MAP2 and successful reexpression of the nuclear ICD was confirmed via Flag staining and evaluating nuclear localization of the Flag-tagged ICD (Fig. 9*A*, right). Consistent with the results shown in Figure 3, we found that DAPT treatment significantly disrupted dendritic growth, but this effect was abolished by reexpression of the nuclear Nrg1 ICD. Importantly, in cultures treated with the AAV-ICD, neurons that lacked detectable levels of the Nrg1 ICD in the nucleus were not rescued from the effects of DAPT [Fig. 9*A*, right, and Fig. 9*C*; Kruskal–Wallis test, *p* = 0.0001; KW = 22.94; DAPT vs DAPT + AAV-ICD, *p* = 0.03; DAPT vs Veh + AAV-ICD, *p* = 0.004; DAPT + AAV-ICD vs DAPT + AAV-ICD (nonexpressing), *p* = 0.0045; DAPT + AAV-ICD (nonexpressing) vs Veh + AAV-ICD, *p* = 0.0007; DAPT vs DAPT + AAV-ICD (nonexpressing), *p* > 0.9999].

The dendritic defects originating from γ-secretase hypofunction can be rescued in a cell autonomous manner by reexpression of the nuclear Nrg1 ICD; however, we posit that the endogenous source of this nuclear ICD is a consequence of axon–dendrite interactions during synaptogenesis (Fig. 4).

#### Nrg1 V_321_L mutant mice show sensorimotor gating deficits and a hyperresponsive auditory startle reflex

**Figure 10.**
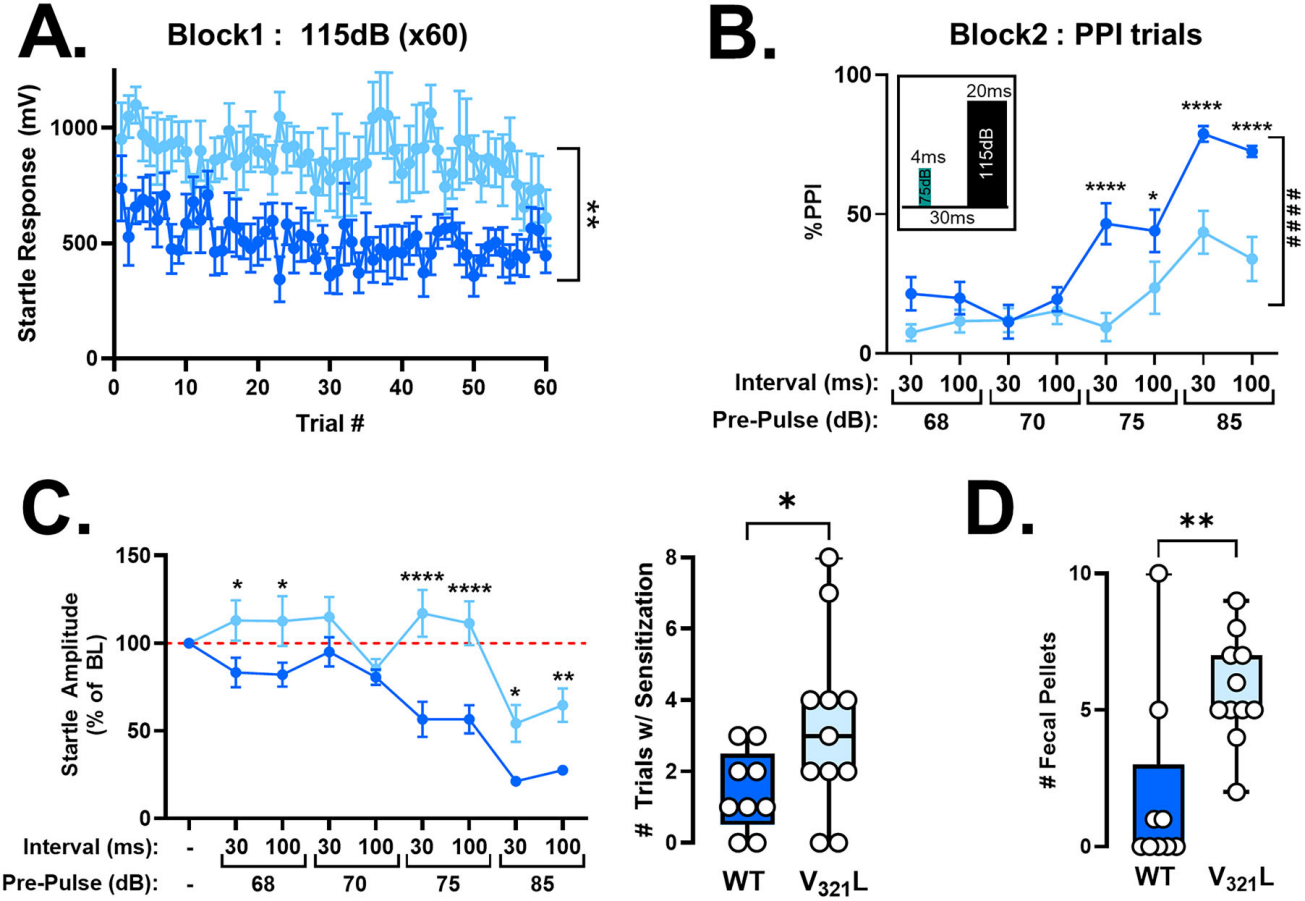
V_321_L mutant mice show sensorimotor gating deficits. ***A***, Startle responses during Block 1 trials, comprised of 60 startle stimuli (115 dB). Neither WT (dark blue) nor V_321_L (light blue) mice showed significant habituation of the startle response during the testing session. However, V_321_L mice showed a significantly larger startle response (**); two-way RM ANOVA trial × genotype, *p* = 0.40; trial, *p* = 0.27; genotype, *p* = 0.005; *F*_(1,18)_ = 10.45. Data represented as mean ± SEM. *N* = 9 mice (WT) and 11 mice (V_321_L). ***B***, V_321_L mice showed significantly lower PPI compared with WT mice; two-way RM ANOVA trial type × genotype, ^####^*p* < 0.0001; *F*_(7,126)_ = 5.387; trial type, *p* < 0.0001; genotype, *p* = 0.0026. Multiple comparisons (WT vs V_321_L; Bonferroni-corrected): 75 dB-30 ms–115 dB, ****p < 0.0001; 75 dB-100 ms–115 dB, *p = 0.02; 85 dB-30 ms–115 dB, ****p < 0.0001; 85 dB-100 ms–115 dB, ****p < 0.0001. Data represented as mean ± SEM. *N* = 9 mice (WT) and 11 mice (V_321_L). Inset shows a schematic of a representative PPI trial consisting of a 4 ms 75 dB prepulse and a 20 ms 115 dB startle stimulus separated by 30 ms. The prepulse intensities and intervals between prepulse and startle pulse were varied; different combinations are displayed on the *x*-axis. ***C***, V_321_L mice showed sensitization of the startle response. Left, Plot shows startle amplitude normalized to baseline startle response (BL), which is the average of the startle response during startle alone trials in Block 2. Red dashed line delineates 100%, representing no suppression of startle response. An increase beyond baseline startle (red dashed line) indicates a sensitized response; two-way RM ANOVA trial × genotype, *p* < 0.0001; *F*_(8,144)_ = 4.977; trial type, *p* < 0.0001; genotype, *p* = 0.009. Data represented as mean ± SEM. The 9/11 V_321_L and 4/9 WT mice showed sensitization on at least one of the trials. Right, V_321_L mice showed sensitized startle responses on more trials than WT mice; Welch’s *t* test (two-tailed), *p* = 0.05; *t* = 2.147; df = 14.39. *N* = 9 mice (WT) and 11 mice (V_321_L). ***D***, V_321_L mice showed increased defecation compared with WT mice consistent with a more anxious behavioral state; Mann–Whitney test, *p* = 0.006; *U* = 15. *N* = 9 mice (WT) and 11 mice (V_321_L).

Orthologs of SCZ-associated genes were found to be dysregulated in the V_321_L DG (Extended Data Table 5-3). The V_321_L mutation in Nrg1 was discovered using a family-based association test where it was found to be overtransmitted to offspring with psychosis and SCZ (Walss-Bass et al., 2006). Additionally, both Type III Nrg1 heterozygous mice and mice deficient in γ-secretase show sensorimotor gating deficits, an endophenotype of psychotic disorders (Chen et al., 2008; Dejaegere et al., 2008). Thus, we asked whether the V_321_L mice showed sensorimotor gating deficits by assessing PPI of the auditory startle reflex. We first subjected WT and V_321_L mice to 60 trials of a 115 dB startle stimulus to measure the startle amplitude and assess habituation of the startle response (Fig. 10*A*). We found that neither WT nor V_321_L mice showed significant habituation of the startle reflex (Fig. 10*A*; two-way RM ANOVA; effect of trial number, *p* = 0.3). However, V_321_L mice consistently showed significantly larger startle responses compared with WT mice (Fig. 10*A*; two-way RM ANOVA; effect of genotype, *p* = 0.005). Next, the mice were subjected to 160 trials consisting of startle stimulus delivered alone or preceded by a prepulse of varying amplitudes and varying delays (Fig. 10*B*). WT mice showed significantly higher PPI on trials consisting of either 75 dB or 85 dB prepulses compared with either 68 dB or 70 dB trials, neither of which elicited PPI (Fig. 10*B*; two-way RM ANOVA; Bonferroni-corrected, *p* < 0.05). V_321_L mice only showed statistically significant increase in PPI on 85 dB trials compared with that on other trials; however, PPI was significantly impaired compared with WT mice (Fig. 10*B*; two-way RM ANOVA; genotype × trial type, *p* < 0.0001). Additionally, V_321_L mice showed significantly higher startle responses on the nonprepulse trials interspersed throughout the PPI trial block (WT vs V_321_L, *p* = 0.03; Welch’s *t* test, *t* = 2.362; df = 15.91). Upon comparing startle responses during nonprepulse trials to those with prepulses, we noticed a general trend toward sensitization of the startle response in the V_321_L mice compared with that in WT mice (Fig. 10*C*, left). We found that a larger proportion of V_321_L mice show sensitization on a greater number of trials irrespective of prepulse intensity, whereas WT mice only ever showed sensitization during the mild prepulse trials (68–70 dB) and always showed startle suppression on the relatively stronger prepulse trials (75 and 85 dB; Fig. 10*C*; right WT vs V_321_L, *p* = 0.05; Welch’s *t* test, *t* = 2.147; df = 14.39).

We also measured number of fecal pellets as a measure of anxiety (Hall, 1934). Mice were handled and acclimated to the testing environment for 3 d and then subjected to the PPI testing protocol on 2 consecutive days. WT mice showed little to no defecation during the final PPI testing; however, V_321_L mice continued to defecate at significantly higher rates than WT mice indicating a potentially higher anxiety-like behavioral state (Fig. 10*D*; WT vs V_321_L, *p* = 0.006; Mann–Whitney rank sum test, *U* = 15).

#### Dysregulation of a functionally connected SCZ-susceptibility gene network in the V_321_L DG

**Figure 11.**
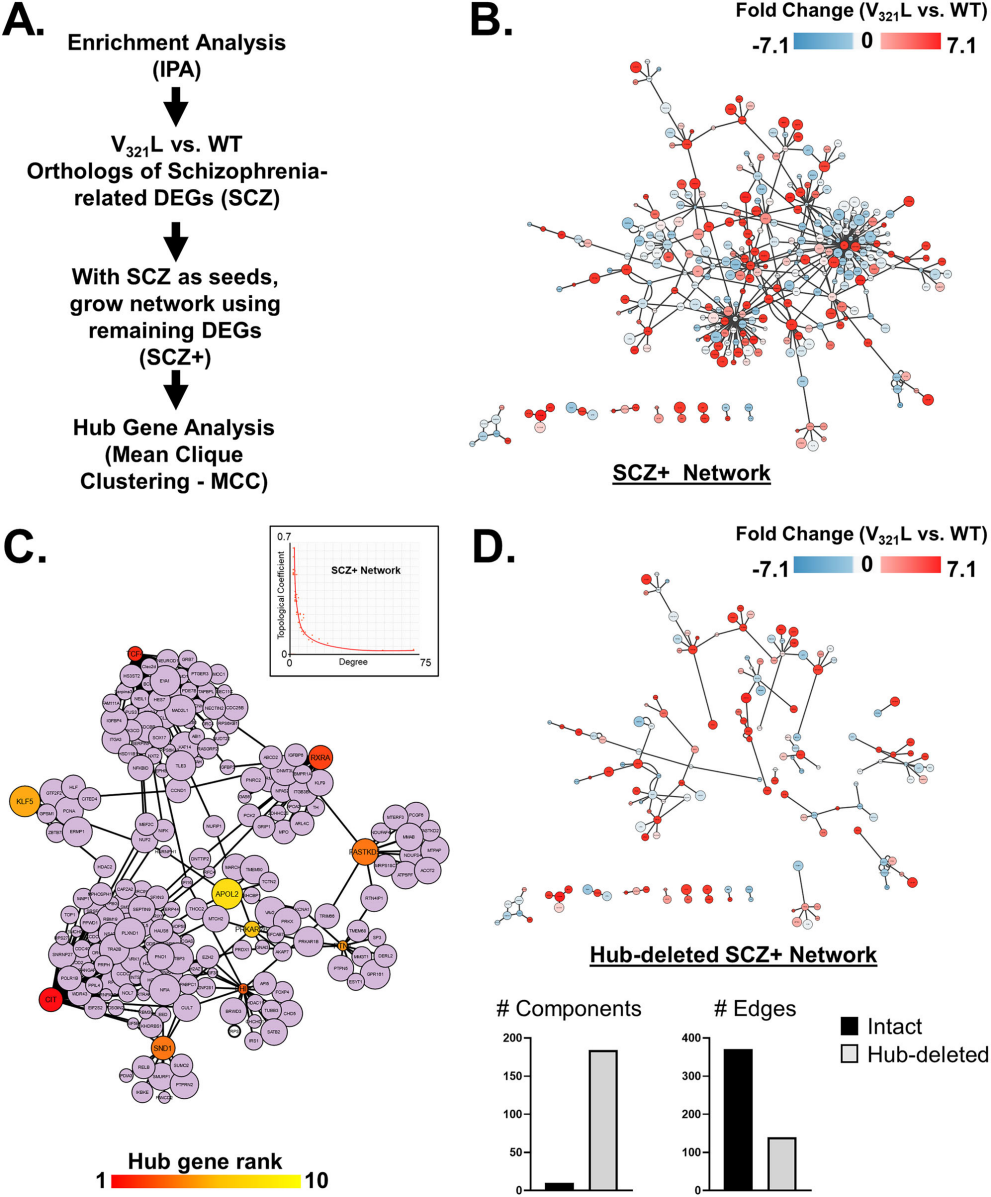
Dysregulation of a SCZ-susceptibility gene network in the V_321_L DG. ***A***, Workflow outlining the generation and analysis of a SCZ-susceptibility gene network. ***B***, Orthologs of genes associated with SCZ spectrum disorders (Fig. 5*D*, IPA analysis) were found to be dysregulated in the V_321_L DG (Extended Data Table 5-3). A network was grown by seeding SCZ-related dysregulated genes (V_321_L vs WT) along with the remaining set of DEGs between V_321_L and WT DG, in IPA. We labeled this network – SCZ±. Node colors represent FC in expression (−7.1 blue to 7.1 red) according to the heatmap. This network was subjected to hub gene analysis using the MCC method. Also see Extended Data Tables 11-1 and 11-2. ***C***, Network display of 10 hub genes identified by MCC analysis of the SCZ+ network, along with their first-degree neighbors (lavender colored nodes) shows a hub-spoke connectivity with SCZ-related genes forming hubs. Hub node color reflects MCC rank. The inset shows a plot showing the relationship between the degree and topological coefficient of every node in the SCZ+ network. The inverse relationship indicates the presence of potential hubs in the network. ***D***, Top, Hub genes identified in panel ***C*** were deleted from the SCZ+ network, disconnected nodes were removed from the network, and the resulting network is displayed. Bottom, Quantification of number of connected components and edges in the SCZ+ network and Hub-deleted SCZ+ network. Node colors represent FC in expression (−7.1 blue to 7.1 red) according to the heatmap.

Transcriptomic enrichment analyses combined with empirical studies shown thus far strongly implicated the Nrg1 ICD in regulating a functionally connected network of genes involved in neuronal development. The enrichment of orthologs of SCZ-associated genes within the dysregulated genes in the V_321_L DG raised the possibility that the SCZ-associated genes might also function within this larger network of genes regulating neuronal development. To gain further insight into how SCZ-associated DEGs in the V_321_L DG might regulate neuronal development, we first asked if the SCZ-associated DEGs in the V_321_L DG functionally interact with the larger set of all the other DEGs in the V_321_L DG. To do this, we created a merged list of dysregulated genes in the V_321_L DG whose human orthologs are associated with psychosis, SCZ, sporadic SCZ, or schizoaffective disorder, under the umbrella term of “SCZ spectrum disorders” yielding a total of 67 genes (Fig. 11*A*; enrichment *p* value = 5 × 10^−7^; Extended Data Table 11-1). We then grew a network (see Materials and Methods) by providing the rest of the significantly DEGs in the V_321_L DG seeking to find genes in our dataset that have not been explicitly identified as SCZ-associated but might have direct functional interactions with dysregulated SCZ-annotated genes. This resulted in a network with 322 nodes and 371 edges, which we called the “SCZ+” network. Forty-nine of the 67 SCZ genes were incorporated into this SCZ+ network (Fig. 11*B*, right; Extended Data Table 11-1). Thus, the bulk (73%) of the SCZ-associated DEGs do interact within a larger network of DEGs, which as previously shown, are involved in various neurodevelopmental processes (Fig. 5, Extended Data Table 5-2). Next, we analyzed the topological properties of this network to derive further insight into the nature of these interactions aiming to further distill the role SCZ genes might play within this network.

10.1523/JNEUROSCI.0063-24.2024.t11-1 Table 11-1 **List of genes part of the SCZ + network. (Top)** 49/67 SCZ DEGs that incorporate into SCZ + network. **(Bottom)** 271 DEGs that are added to the SCZ + network. Fold change in gene expression and ANCOVA target corrected p-values are reported alongside. Download Table 11-1, XLSX file.

10.1523/JNEUROSCI.0063-24.2024.t11-2 Table 11-2 **Gene Ontology (GO) analyses for genes in the SCZ + network. (Top)** GO Biological process and SynGO enrichment for 49/67 SCZ DEGs that incorporate into SCZ + network. **(Bottom)** GO Biological process and molecular function of 271 DEGs that are added to the SCZ + network. Download Table 11-2, XLSX file.

Analysis of the expanded SCZ-susceptibility network (SCZ+) implicated a “hub and spoke” topology. Hub nodes interacted preferentially with a few other nodes as opposed to each other (i.e., the degree of a node and its topological coefficient were inversely related; Fig. 11*C*, inset). We identified 10 hub nodes using maximal clique centrality (MCC), 9 of which were part of the primary SCZ-susceptibility network (Fig. 11*C*). The 10 hubs shared no edges with each other thereby creating 10 modules [red (higher rank) to yellow (lower rank) MCC top 10 nodes]. We visualized the first-degree neighbors of these hub genes and found that each of the hub genes coordinated its own mini network as predicted from the network topology (Fig. 11*C*). To validate the status of the hub genes as hubs within the SCZ+ network, we reconstructed the SCZ+ network shown in Figure 11*A* after removing the hub genes from the network. Exclusion of these 10 hub genes resulted in fragmentation of the network indicated by the drastic increase in the number of components and decrease in the number of edges (Fig. 11*D*). Thus, the SCZ genes not only interact with a larger network of dysregulated genes, but a subset of them (9 out of the original 67) are essential for organizing the network.

Next we performed gene ontology analyses on the 49 SCZ genes and the 271 DEGs that became part of SCZ+ network. The SCZ genes were enriched for functions related to synaptic transmission (adj. *p* = 4.02 × 10^−8^) and were enriched for genes whose products are localized to synaptic membranes (SynGO, adj. *p* < 0.01). Intriguingly, none of the hub genes (90% of which were part of the original SCZ-associated gene list) were associated with synaptic transmission. The 271 DEGs that comprised the SCZ+ network were also enriched for the annotation–TR (adj. *p* = 0.02; driven by 52 of the 271 genes) and were enriched for genes whose products are localized to the nucleus (adj. *p* = 0.00002; Extended Data Table 11-2). These data show that while SCZ-associated DEGs are enriched for genes involved in synaptic function, hubs that embed these core genes into cellular signaling networks are not obvious mediators of synaptic function.

#### Nrg1 nuclear back signaling regulates functionally related gene modules

**Figure 12.**
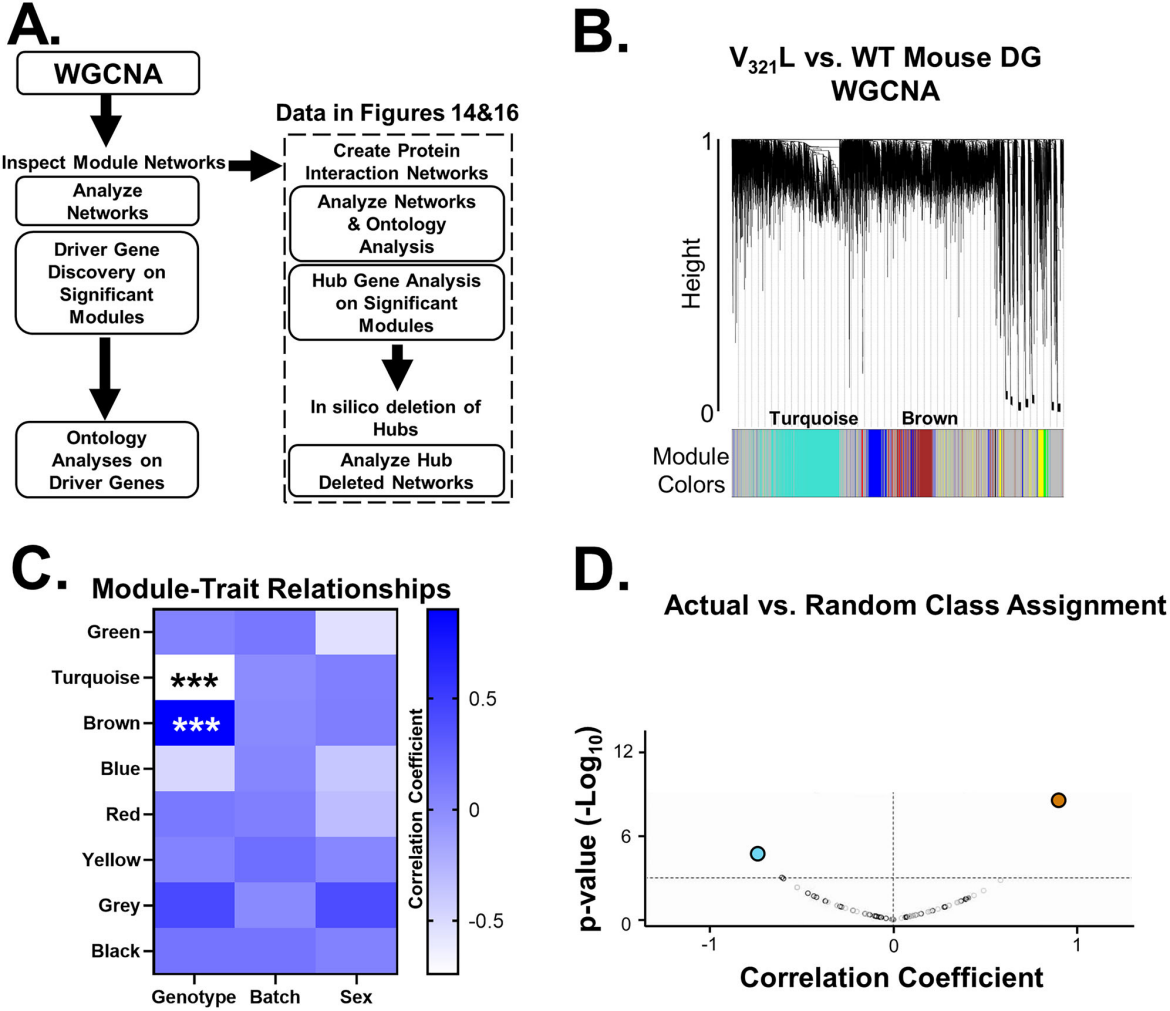
WGCNA of V_321_L mouse DG. ***A***, Flowchart outlining the analysis strategy following WGCNA module discovery. PI networks constructed from WGCNA modules are shown in Figure 14. ***B***, Module detection (WGCNA) via dynamic tree cut from the WT versus V_321_L DG transcriptome. The detected modules are represented by colors below the dendrogram. The two statistically significant modules (turquoise and brown) are labeled. ***C***, The heatmap showing module–trait relationships between detected modules in panel ***A*** and the V_321_L genotype, sequencing batch (technical factor), and sex of the animal. The color indicates the correlation coefficient according to the legend. Only two modules were statistically significantly associated with genotype and not with sequencing batch. We dubbed the turquoise module as the mouseDOWN module (*p* = 0.009) and the brown module as the mouseUP module (*p* = 0.0002) for a more intuitive nomenclature. None of the modules were associated with chromosomal sex of the mice. ***D***, Permutation analysis of module discovery–gene memberships were permuted 40 times. None of the permuted modules had equivalent correlation coefficients and statistical significance as the observed modules. The gray open circles represent permuted turquoise modules and black open circles represent permuted brown modules.

TR analysis showed that DEGs in the V_321_L DG can be regulated by a small set of TRs (e.g., REST, PRC2, E2F4, etc.). TR analysis also showed that DEGs regulated by the same regulators were functionally related (Extended Data Tables 5-4, 5-5). This result raises the possibility that the Nrg1 ICD transcriptionally regulates functionally related genes. To investigate this, we performed WGCNA to identify gene coexpression modules associated with the V_321_L mutation (Fig. 12*A*,*B*). WGCNA identified two modules in which expression of genes was statistically significantly correlated [brown module, mouseUP module; *p* = 0.0002; corr.coefficient (LL genotype) = 0.9] or anticorrelated [turquoise module, mouseDOWN module; *p* = 0.009; corr.coefficient (LL genotype) = −0.74] with the mutant genotype (Fig. 12*C*). We performed permutation analyses to validate the correlation coefficients and *p* values and found that randomized module membership (MM) could not achieve the same values as observed (Fig. 12*D*).

**Figure 13.**
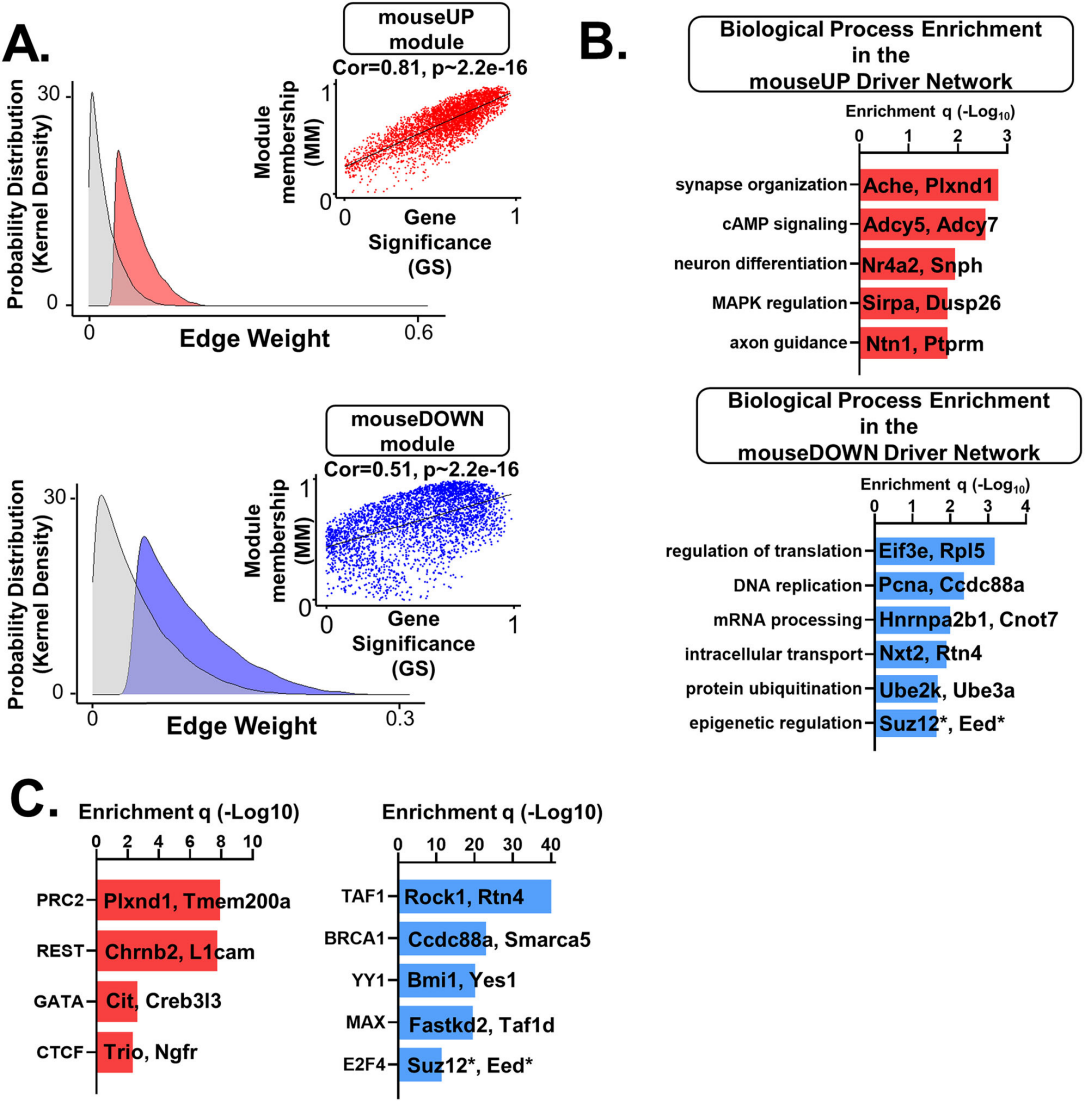
Analysis of driver genes and driver gene networks from the V_321_L mouse WGCNA. ***A***, Deletion of selected driver genes from the WGC network alters the weight distribution of the network indicating their significance within the network. Top, MouseUP [intact (red) vs driver deleted (gray), Kolmogorov–Smirnov, *D* = 0.05; *p*∼2.2 × 10^−16^]. Bottom, MouseDOWN [intact (blue) vs driver deleted (gray), Kolmogorov–Smirnov *D* = 0.59 *p*∼2.2 × 10^−16^]. Insets show correlation between GS and MM for both mouseUP and mouseDOWN modules. Correlation analysis reveals a strong relationship between GS and MM for the mouseUP module (Pearson's corr., 0.81; *p* = 2.2 × 10^−16^) indicating their significance to the dysregulated V_321_L DG transcriptome and importance within the mouseUP module. Correlation for the mouseDOWN module was not as strong but still revealed a significant relationship between GS and MM (corr. 0.5; *p* = 4.2 × 10^−16^); driver genes were selected as ones having the highest MM (top 95th percentile). ***B***, Ontology analysis showing biological processes enriched within the driver gene network. Top (red), Biological processes implicated in the mouseUP driver gene network. Bottom (blue), Biological processes implicated in the mouseDOWN driver gene network. Asterisks highlight the presence of PRC2 core components in the mouseDOWN module. ***C***, Predicted TRs for the driver genes from the ChEA and ENCODE databases reveal PRC2 as a strong candidate in regulating genes in the mouseUP module and TAF1 for the mouseDOWN module. Asterisks highlight the presence of PRC2 core components in the mouseDOWN module predicted to be regulated by E2F4.

Next, we asked whether the genes whose expression was significantly associated with the mutant genotype were also important members of these modules as this would warrant further investigation into the gene networks that comprise these modules. To do this, we examined the relationship between MM (coexpression between each gene in a module with the module eigengene) and gene significance (GS) to the mutant genotype (correlation of gene expression with genotype; Fig. 13*A*, insets). Both modules showed significant correlation between these two metrics indicating that the genes associated with (dysregulated by) the mutant genotype were proportionately important to the networks within each module (Fig. 13*A*, top (inset), mouseUP, corr = 0.79; *p* < 0.0001; Fig. 13*A*, bottom (inset), mouseDOWN, corr = 0.53; *p* < 0.0001). We identified genes with the 95th percentile of MM scores within each module as putative driver genes for the network. These genes were then removed from the network. Since the network was weighted, we predicted that loss of driver genes would bias the network to lower weighted edges thereby destabilizing the network. Indeed, loss of the identified driver genes created a significant left shift in the weight distributions in both modules (Fig. 13*A*, top, mouseUP, KS test, *p* = 2.2 × 10^−16^; and bottom, mouseDOWN, KS test *p* = 2.2 × 10^−16^).

To understand what functions, if any, were overrepresented by the driver genes, we performed gene ontology analyses on the driver genes and found that the mouseUP driver genes were enriched for predominantly axonal biased functions—synapse organization, cAMP signaling, and axon guidance, along with others such as neuronal differentiation and negative regulation of MAPK signaling (Fig. 13*B*, top). The mouseDOWN driver genes were largely enriched for ribostatic and proteostatic functions (Fig. 13*B*, bottom). Next, given that these genes were identified through a coexpression analysis, we sought to identify potential regulatory logic to understand the coregulation of these driver genes. Using the ChEA and ENCODE databases, we assessed enrichment for TRs that could regulate the driver genes. As with the thresholded upregulated genes (Extended Data Table 5-4), we once again observed a signature of regulation by PRC2 and REST along with GATA and CTCF for the mouseUP driver genes (Fig. 13*C*, left). The mouseDOWN driver genes were enriched for TAF1-regulated genes and genes regulated by BRCA1, YY1, MAX, and E2F4 (Fig. 13*C*, right). Intriguingly, two core components of the PRC2, *Suz12* and *Eed*, were within the E2F4-regulated gene list, which were downregulated in V_321_L DG compared with WT DG.

**Figure 14.**
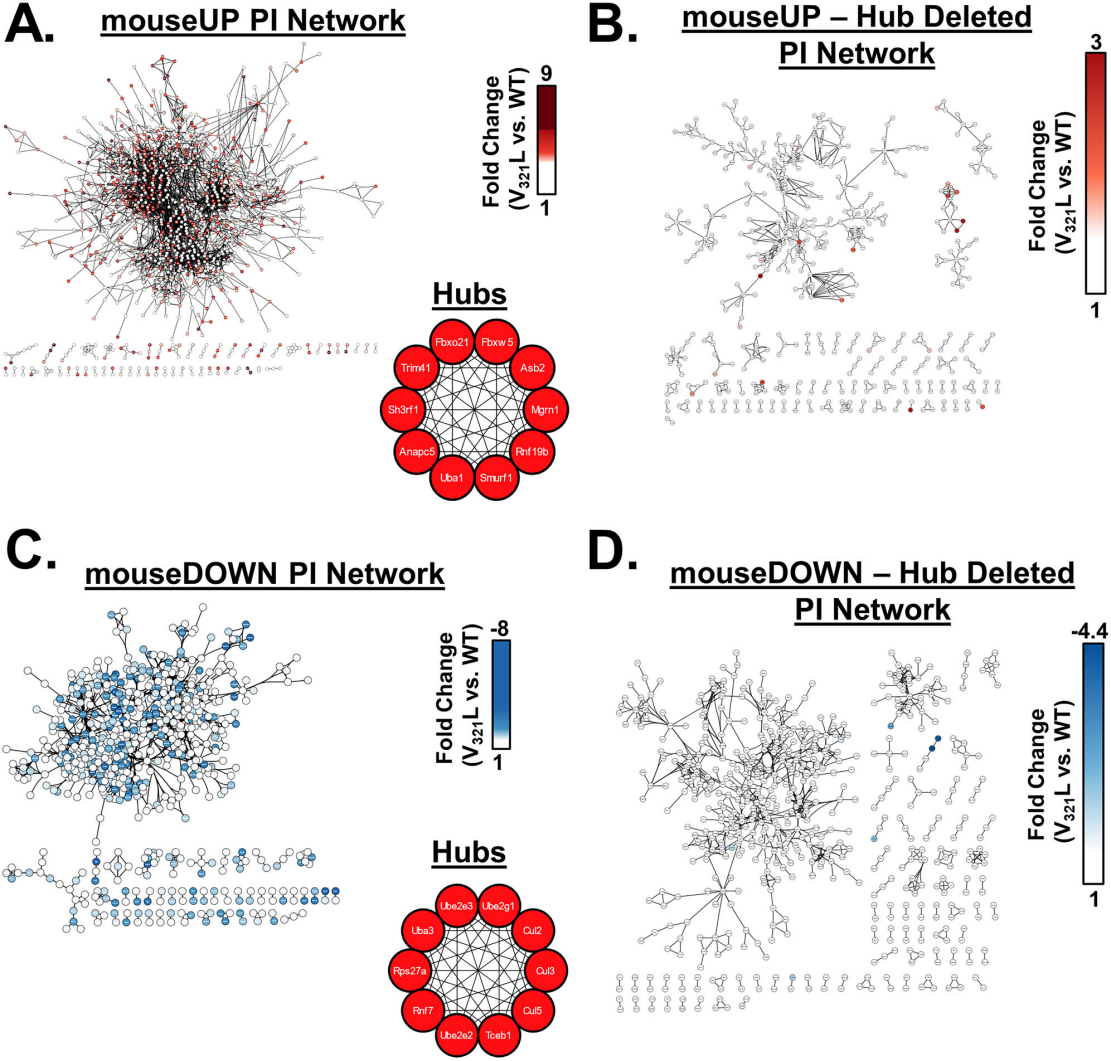
Ubiquitin ligases maintain network cohesion in PINs of V_321_L mouse WGCNA modules. PI networks were constructed (see Materials and Methods for details) from WGCNA modules. Resulting networks were densely interconnected. Nodes are color coded according to FC in expression (V_321_L vs WT) indicated by the heatmaps in the respective graphs. Edges were bundled for clarity. ***A***, mouseUP PIN, PIN from genes positively correlated (upregulated) with the V_321_L genotype (1,910 nodes; average node degree, 3.2; PPI enrichment, *p* = 3.56 × 10^−13^). Node color legend: FC 1 white to 9 deep red. (Inset) mouseUP PIN was analyzed using MCC to find a ranked list of hub proteins within the network. The top 10 hubs for this network were all coranked as a Rank 1 (red) and were ubiquitin ligases or accessory factors. ***B***, Hub genes embed the top upregulated genes in the V_321_L DG in the mouseUP PIN. The panel shows a PIN from genes positively correlated (upregulated) with the V_321_L genotype after the MCC hub genes were removed from the network and disconnected nodes were removed (479 nodes; average node degree, 3.14). Node color legend: FC 1 white to 3 deep red. Also see Extended Data Table 14-1. ***C***, mouseDOWN PIN: PIN from genes negatively correlated (downregulated) with the V_321_L genotype (1,870 nodes; average node degree, 4.7; PI enrichment, *p* < 1.0 × 10^−16^). Node color legend: FC −8 blue to 1 white. Inset, mouseDOWN PIN was analyzed using MCC to find a ranked list of hub proteins within the network. The top 10 hubs for this network were all coranked as a rank 1 (red) and were ubiquitin ligases or accessory factors. ***D***, Hub genes embed the most downregulated genes in the V_321_L DG in the mouseDOWN PIN. The panel shows a PIN from genes negatively correlated (downregulated) with the V_321_L genotype after the MCC hub genes were removed from the network and disconnected nodes were removed (577 nodes; average node degree, 4.38). Node color legend: FC −4.4 blue to 1 white. Also see Extended Data Table 14-1.

Genes clustered within a WGCNA module have been shown to be functionally related (Kos et al., 2018). Thus, we asked whether the genes with correlated expression in each module encode proteins that have functional interactions. We accomplished this using the STRING protein interaction (PI) database (Szklarczyk et al., 2023) to build PI networks (PINs) from the WGCNA modules. Using stringent criteria (see Materials and Methods) we extracted a PIN using genes within the WGCNA modules (Fig. 14). We found that the protein products of genes within the WGCNA modules had highly significant functional interactions forming densely interconnected networks (Fig. 14*A*, mouseUP module, 1,136 nodes; average node degree, 8.2; PI enrichment *p* value = 3.56 × 10^−13^; Figure 14*C*, mouseDOWN module, 712 nodes, average node degree, 15; PI enrichment *p* value < 1.0 × 10^−16^).

10.1523/JNEUROSCI.0063-24.2024.t14-1 Table 14-1 **Network statistics for hub gene deletion from mouseUp and mouseDown PINs.** Network analysis showing decomposition of the PINs generated from WGCNA modules. Download Table 14-1, XLSX file.

To gain insight into the nature of these functional interactions, we analyzed the networks as undirected graphs. We identified hub nodes based on their MCC (Chin et al., 2014). The highest MCC ranked genes in the mouseUP and mouseDOWN modules were members of ubiquitin ligase complexes indicating that a functionally related group of hub genes was disrupted within these modules (Fig. 14*A*,*C*, insets). To probe the significance of these hub genes in maintaining integrity of the larger network and validate their hub status, we removed them from the network and reanalyzed the remaining genes for interactions in STRING for both mouseUP and mouseDOWN modules. Deletion of the MCC hub genes resulted in reduced connectivity within the network and disintegration of the network (Extended Data Table 14-1), thereby supporting their role as hub genes; the hub-deleted network resulted in loss of almost all the highest DEGs between V_321_L and WT DG as evidenced by the loss of darker colored nodes from the networks (Fig. 14*B*,*D*).

Thus, WGCNA revealed that loss of Nrg1 nuclear back signaling results in highly correlated dysregulation of functionally related modules of genes.

#### Molecular changes in the mouse V_321_L mutant DG show similarity with those observed in DG samples from humans with SCZ

The bulk of the genetic variation associated with SCZ is regulatory in nature (eQTLs; Ripke et al., 2014). However, available databases do not yet provide clear mechanisms for how these variants might influence biological processes that underlie SCZ pathology. Our network analyses and ontology analyses on the V_321_L mouse DG transcriptome allowed us to uncover latent regulatory structure within the transcriptomic data. We next sought to apply the same network analysis pipeline to anatomically matched human DG transcriptomic data from people with SCZ to ask which, if any, features uncovered in the analysis of the V_321_L mouse DG might be represented in human pathology.

**Figure 15.**
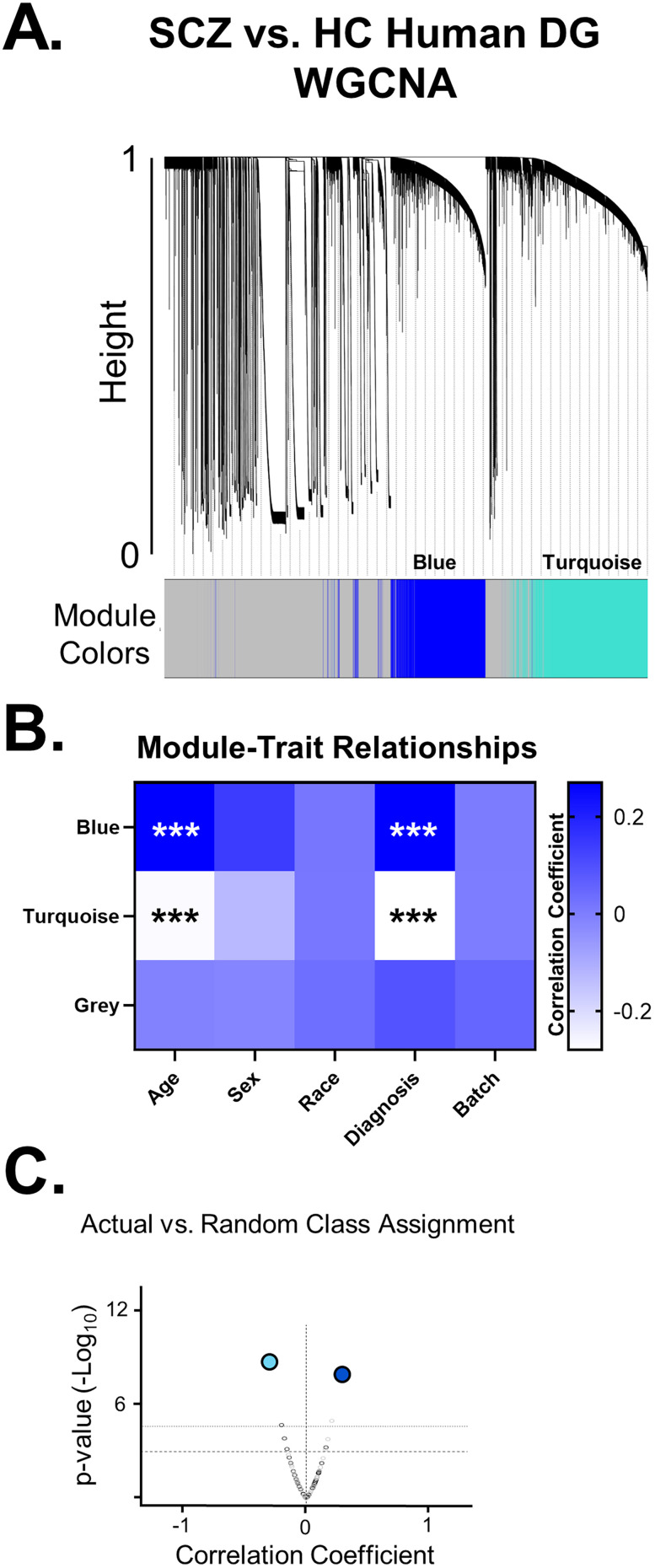
WGCNA of human SCZ DG transcriptomes. ***A***, Module detection (WGCNA) via dynamic tree cut from the healthy control versus SCZ DG transcriptome. The detected modules are represented by colors below the dendrogram. The two statistically significant modules (turquoise and blue) are labeled. ***B***, Heatmap showing module–trait relationships between detected modules in panel ***A*** and SCZ diagnosis, biological factors such as age and sex of the subjects, and race of the subjects and sequencing batch. The color indicates the correlation coefficient according to the legend. Only two modules were statistically significantly associated with diagnosis and age but not with sequencing batch or race. We dubbed the turquoise module as the humanDOWN module (module × diagnosis, *p* = 0.0002; module × age, *p* = 0.0005) and the blue module as the humanUP module (module × diagnosis, *p* = 0.0004; module × age: 0.0005) for a more intuitive nomenclature. ***C***, Permutation analysis of module discovery–gene memberships were permuted 40 times. None of the permuted modules had equivalent correlation coefficients and statistical significance as the observed modules. The gray open circles represent permuted turquoise modules, and black open circles represent permuted blue modules.

We performed WGCNA using published RNA-Seq data from microdissected DG GCL from healthy controls and people with SCZ (93 controls and 75 SCZ subjects, 119 males and 49 females; Jaffe et al., 2020; Fig. 15). WGCNA identified two modules wherein gene expression was significantly (positively or negatively) correlated with SCZ diagnosis (Fig. 15*A*,*B*). We renamed the module colors for clarity to parallel the data from the mutant mouse DG [blue module, humanUP module; *p* = 0.0004; corr.coefficient (SCZ) = 0.3; corr.coefficient *p* = 0.0005; turquoise module, humanDOWN module; *p* = 0.0002; corr.coefficient (SCZ) = −0.3; *p* = 0.0005] and not with sequencing batch, race, or sex of the individuals (Fig. 15*B*). We also performed permutation analysis to validate the discovered modules as we did for the mouse DG samples and did not find values that matched or exceeded the actual values (Fig. 15*C*).

**Figure 16.**
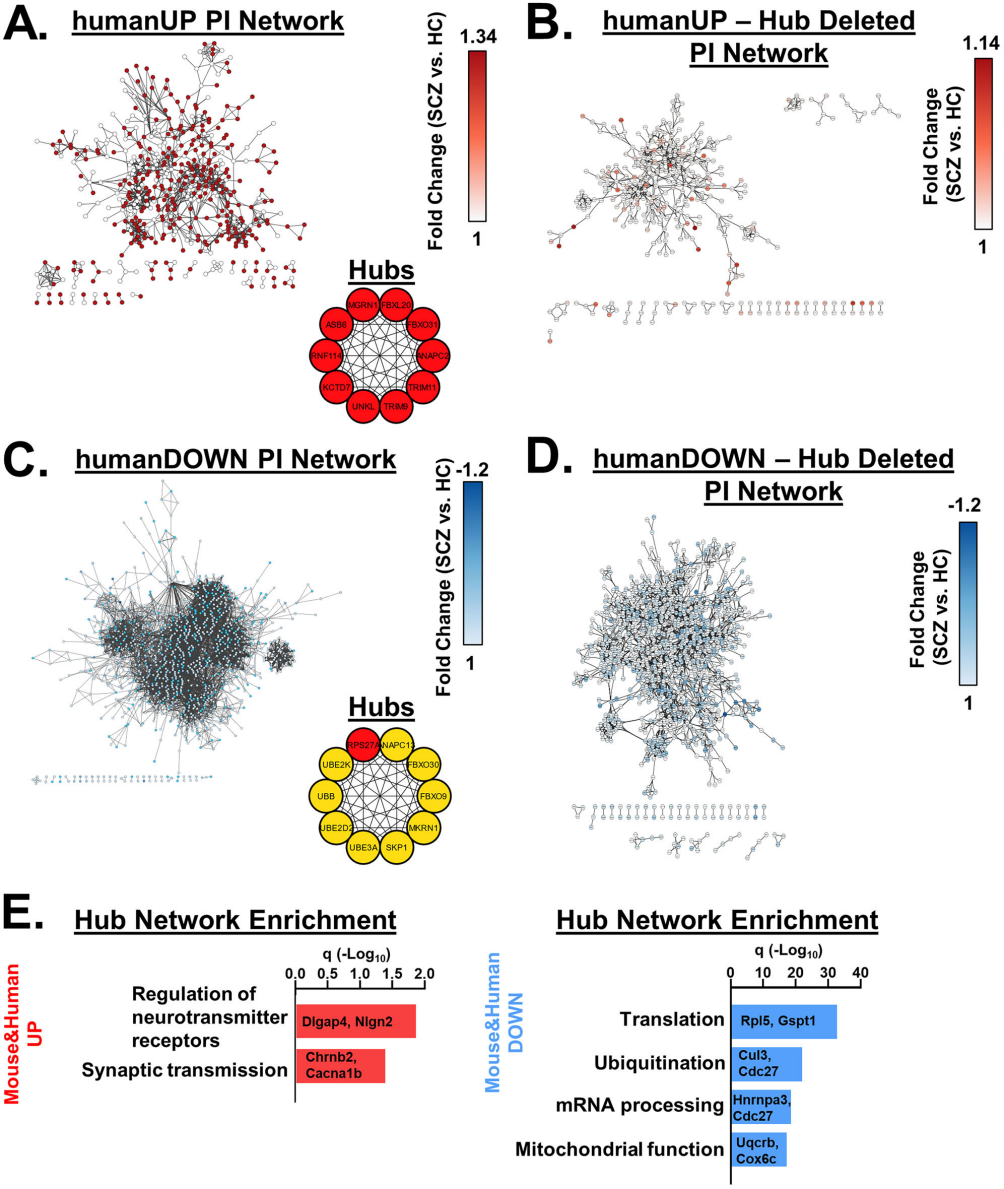
Shared functional molecular risk profile between V_321_L mutant mouse DG and postmortem DG samples of people who were diagnosed with SCZ. ***A***, humanUP PIN: PIN from genes positively correlated (upregulated) with SCZ diagnosis (1,364 nodes; average node degree, 8.96; PI enrichment, *p* = 1.96 × 10^−7^). Node color legend: FC 1 white to 1.14 deep red. Inset, humanUP PIN was analyzed using MCC to find a ranked list of hub proteins within the network. The top 10 hubs for this network were all coranked as a Rank 1 (red) and were ubiquitin ligases or accessory factors. ***B***, Hub genes embed the top upregulated genes in the DG of patient samples in the humanUP PIN. The panel shows a PIN from genes positively correlated (upregulated) with the SCZ diagnosis after the MCC hub genes were removed from the network and disconnected nodes were removed (361 nodes; average node degree, 7.9). Node color legend, FC 1 white to 1.14 deep red. ***C***, humanDOWN PIN: PIN from genes negatively correlated (downregulated) with SCZ diagnosis (1,947 nodes, average node degree, 23.56; PI enrichment, *p* = <1.0 × 10^−16^). Node color legend: FC −1.2 blue to 1 white. Inset, humanDOWN PIN was analyzed using MCC to find a ranked list of hub proteins within the network. The network had one Rank 1 hub gene (*RPS27A*, red), and the other nine were coranked for Rank 10 (yellow). All hub genes were identified as ubiquitin ligases or accessory factors. ***D***, Hub genes embed the top downregulated genes in the DG of patient samples in the humanDOWN PIN. The panel shows a PIN from genes negatively correlated (downregulated) with SCZ diagnosis after the MCC hub genes were removed from the network and disconnected nodes were removed (1,064 nodes; average node degree, 22.47). Node color legend: FC −1.2 blue to 1 white. ***E***, Gene ontology analysis of the up- and downregulated genes that comprised the hub genes identified in both V_321_L mouse and SCZ human PINs, and their first-degree neighbors from their respective networks showed complete overlap of the biological processes predicted to be altered. Examples of common DEGs between species are displayed.

To validate that the human WGCNA modules represented functionally interacting components, we constructed PINs from them (Fig. 16*A*–*C*). We found that the protein products of genes that were part of the human WGCNA modules had highly significant functional interactions (Fig. 16*A*, humanUP module, 489 nodes; average node degree, 8.96; PI enrichment, *p* = 1.96 × 10^−7^; Fig. 16*C*, humanDOWN module, 1,180 nodes, average node degree, 23.56; PI enrichment, *p* = <1.0 × 10^−16^). Similar to the mouse hub gene analysis, the highest ranked hub genes formed networks that are part of ubiquitin ligase complexes (Fig. 16*A*,*C*, insets). Deletion of the human MCC hub genes resulted in loss of almost all the relatively DEGs between SCZ and HC groups as evidenced by the loss of darker colored nodes from the networks (Fig. 16*B*,*D*).

We created hub networks by selecting the first-degree neighbors for the mouse and human hub genes and asked what biological processes were enriched in these core networks. We found a striking 100% overlap between the mouse and human hub network functions. The mouse and human UP hub networks were enriched for genes involved in synaptic transmission and neurotransmitter receptor regulation by synaptic proteins (Fig. 16*E*, left). The mouseDOWN and humanDOWN hub networks were enriched for genes involved in several cellular homeostatic functions such as proteostasis, ribostasis, and mitostasis (Fig. 16*E*, right).

Thus, these data reveal evolutionarily conserved regulatory programs that might regulate expression and/or function of genes that underlie cellular pathology in psychotic disorders. Furthermore, these data highlight the potential for convergence of molecular pathology of rare and common genetic variants associated with SCZ.

## Discussion

Loss of nuclear back signaling in the V_321_L mouse results in transcriptional changes in the DG and alterations to cell cycle dynamics, neurogenesis, neuronal maturation, and SCZ-related gene expression. These changes were accompanied by pronounced sensorimotor gating deficits. Network analyses uncovered latent gene regulatory factors and functional logic of gene coexpression networks dysregulated by the mutation. Comparison of the V_321_L mouse DG transcriptome with human SCZ DG transcriptomes using WGCNA revealed ubiquitin ligases as hubs coordinating large PINs involved in synaptic transmission, RNA processing/trafficking, and mitochondrial function.

### Regulation of adult hippocampal neurogenesis by Nrg1 nuclear back signaling

A complex interplay of cell intrinsic factors and cell–cell interactions orchestrate neural development. The V_321_L mutation in Nrg1 results in pervasive neurodevelopmental alterations in the DG, including reduced neural stem cell proliferation and increased neuronal differentiation (Fig. 7). DEGs in the V_321_L DG involved in neuronal differentiation were predicted to be regulated by the PRC, components of which were found to be downregulated in the V_321_L DG (Extended Data Table 5-5, Fig. 5). Whether the Nrg1 nuclear ICD directly regulates PRC expression and/or occupancy in the genome in a cell intrinsic manner or whether this is a consequence of downstream circuit level changes due to the mutation are currently unclear. Indeed, activity of several neurotransmitters and neuromodulators can regulate NSC proliferation, survival, and differentiation in the DG (Berg et al., 2013). Thus, it is also possible that loss of Nrg1 nuclear signaling in diverse cell types in various brain regions with the potential to regulate DG neurogenesis might have contributed to these observations.

### Balance between back signaling mechanisms: a tight rope for circuit development

Type III Nrg1 KO neurons show stunted growth of axons and dendrites, which was rescued by reexpression of the FL Type III Nrg1 protein (Chen et al., 2010). Type III Nrg1 is unique in that it localizes to the axonal presynapse indicating that the effects of Nrg1 signaling on axon and dendrite growth require axon localized Nrg1 protein (Vullhorst et al., 2017). Axon and dendrite outgrowth are regulated by two distinct Nrg1 functions: Nrg1-dependent axonal growth requires activation of PI3K signaling (local back signaling; Fig. 2*D*), whereas dendrite growth requires nuclear back signaling (Fig. 2*E*). It is likely that these signaling functions are coordinated. PI3K signaling is a membrane-associated signaling pathway, and membrane fractions from V_321_L brains showed higher levels of Nrg1 protein (Fig. 2*B*). It is possible that increased membrane residence time of the V_321_L Nrg1 ICD resulting from decreased proteolysis increases the duration of PI3K signaling and enhances local signaling. Figure 2*D* (as well as Chen et al., 2010) shows trends toward longer axons in V_321_L neurons, and inhibiting GC γ-secretase increased the number of primary neurites that were immunoreactive for axonal neurofilaments (Fig. 4*B*,*D*). Based on these findings, we propose that activation of PI3K in Type III Nrg1 expressing axons supports axonal trafficking as previously shown (Hancock et al., 2008; Zhong et al., 2022). The same stimuli capable of activating local back signaling can also activate γ-secretase cleavage of axonal Nrg1 initiating nuclear back signaling. The nuclear ICD downregulates genes related to early axon growth programs and simultaneous upregulates dendritic maturation gene programs (Extended Data Table 6-2). In this model, the nuclear ICD couples axonal growth and target innervation to dendritic arborization. In cultured hippocampal neurons and in developing GCs in vivo, axon growth precedes dendritic maturation (Dotti et al., 1988; Sun et al., 2013). Balanced local and nuclear Nrg1 back signaling provides one mechanism for coordinating afferent and efferent synaptogenesis within individual neurons.

### How does the Nrg1 ICD regulate gene expression?

The Nrg1 ICD lacks any obvious DNA binding domains; however, it has strong transactivation potential (Bao et al., 2003). The upregulated genes in the V_321_L DG were predicted to be targets of the PRC2, the catalytic subunits (*Eed*, *Ezh2*, and *Suz12*) of which were downregulated in the V_321_L DG. How Nrg1 nuclear signaling regulates the expression and/or function of the PRC2 is not known, but antagonistic interactions between PRC and the SWI/SNF chromatin remodeling complexes are proposed to maintain balance between self-renewal and differentiation of stem cells (Seo and Kroll, 2006; Kadoch et al., 2016; Weber et al., 2021). Interestingly, the Nrg1 ICD was shown to interact with Brm and BAF57, core subunits of the SWI/SNF complex, in cultured neural stem cells (Pirotte et al., 2010). It is possible that the Nrg1 ICD regulates competition between these large remodeling complexes to influence cell fate decisions.

We also noted downregulation of several ribosomal subunit genes and *Cenp* (centromere proteins that couple centromeric chromatin to the kinetochore) genes in the V_321_L DG (Extended Data Table 5-1). The kinetochore genes and genes related to regulation of microtubule dynamics are predicted to be E2F4 target genes (Extended Data Table 5-5). The E2F family of transcription factors can function as both activators and repressors. E2F4 is one of the three canonical E2F repressors (Chen et al., 2009). This implies a potential gain of function of E2F4 in the V_321_L mutant. Repressor E2Fs have been shown to interact with the PRC, and E2F4 accumulates in quiescent cells consistent with its role in maintaining differentiated states (Trimarchi et al., 2001; Cuitiño et al., 2019). Thus, it is possible that both the upregulated and downregulated genes in the V_321_L mice result from disrupting PRC occupancy across the genome.

Our findings suggest that PRCs might be temporally regulated during neuronal development to target various genes associated with a common function. In line with this, the presence of “polycomb domains,” which are clusters of polycomb proteins bound to DNA segments located several megabases from each other but located in the same 3D space, has been demonstrated (Blackledge and Klose, 2021). These domains are coordinated by CTCF–cohesin complexes (Rhodes et al., 2020). Genes dysregulated in the V_321_L mutant that are predicted to be regulated by CTCF–cohesin shared functional overlap with the predicted PRC2 target genes (Extended Data Tables 5-4, 5-5).

### Nrg1 nuclear back signaling and genetic risk for psychosis

Genetic variants that influence gene expression, eQTLs, are thought to comprise a substantial proportion of genetic risk in SCZ (Richards et al., 2012). The cellular mechanisms by which these changes in gene expression influence risk for SCZ are not clear. We found a significant enrichment of genes associated with psychotic disorders to be dysregulated in the V_321_L DG (Extended Data Tables 5-3, 11-1). We examined known interaction networks between these genes and found several modular hubs with hub genes represented by the known SCZ-associated genes. This larger network of genes was enriched for TRs with known regulatory variants associated with SCZ (Fig. 11). These data are in line with predictions from the omnigenic model, wherein core genes/functions are part of densely connected cellular networks of peripheral genes/functions (Boyle et al., 2017). Additionally, since the core genes involved in synaptic transmission can be accessed by many peripheral genes with cellular homeostatic functions, our data also inform potential mechanisms explaining how peripheral genes might have a cumulative effect on heritability of SCZ. Intriguingly, while hub genes identified in our SCZ+ network are not obvious mediators of synaptic function (Fig. 11), half of them have been shown to be part of the hippocampal synaptic proteome in both rodents and humans (Koopmans et al., 2018). Strikingly, we observed a very similar profile and high functional overlap when we performed additional network analyses on a published dataset of human SCZ and healthy control DG GCL transcriptomes (Jaffe et al., 2020). In both V_321_L mouse and SCZ human networks, ubiquitin–proteasome system (UPS)-associated genes formed the hubs (Figs. 14, 16). The high degree of functional overlap between the genetic dysregulation of the V_321_L mouse and human SCZ DG samples indicates that there might indeed be convergence between risk factors at the gene regulatory level since it is unlikely that many of the patients whose DG RNA-Seq data were used carrying the V_321_L mutation in Nrg1 (∼0.2% of the people would be predicted to be homozygous for this mutation). Future studies examining gene regulatory network overlaps between various genetic and environmental models of SCZ risk could test for such convergence.

#### Concluding remarks

Psychiatric disease risk variants across various disorders aggregate in signaling pathways involved in protein modification such as the UPS and histone modifications (O’dushlaine et al., 2015). We found that disease-associated pathways can be regulated by a common set of TRs such as the PRCs. We propose that these regulators can in turn be regulated by specific neurodevelopmental signaling networks. The combination of TRs impinging upon gene networks within “risk-associated pathways” and intersecting developmentally regulated signaling events might provide specificity for specific disorders and offer a framework to understand how environmental factors interact with genetic risk associated with disease.

## Data Availability

Plasmids used in this study are available on request. The data generated in this publication have been deposited in NCBI’s Gene Expression Omnibus (Edgar et al., 2002) and are accessible through GEO Series Accession Number GSE192869 (https://www.ncbi.nlm.nih.gov/geo/query/acc.cgi?acc=GSE192869) and GSE268856 (https://www.ncbi.nlm.nih.gov/geo/query/acc.cgi?acc=GSE268856).

The code is available at https://github.com/RajNINDS/V321L_DG.
